# A General Method to Develop Highly Environmentally Sensitive Fluorescent Probes and AIEgens

**DOI:** 10.1002/advs.202104609

**Published:** 2021-12-19

**Authors:** Rong Miao, Jing Li, Chao Wang, Xuefeng Jiang, Ying Gao, Xiaoling Liu, Dan Wang, Xin Li, Xiaogang Liu, Yu Fang

**Affiliations:** ^1^ Laboratory of Applied Surface and Colloids Chemistry Ministry of Education School of Chemistry and Chemical Engineering Shaanxi Normal University Xi'an 710062 P. R. China; ^2^ Fluorescence Research Group Singapore University of Technology and Design Somapah Road Singapore 487372 Singapore; ^3^ College of Pharmaceutical Sciences Zhejiang University 866 Yuhangtang Street Hangzhou 310058 P. R. China; ^4^ Present address: Jilin Engineering Normal University Kaixuan Road, 3050 Changchun 130052 P. R. China

**Keywords:** AIEgens, environmental sensitivity, fluorescent probe, *N*‐methylpyrrole, twisted intramolecular charge transfer (TICT)

## Abstract

Environmentally sensitive fluorescent probes (including AIEgens) play pivotal roles in numerous biological studies. Many of these functional materials are developed based on the twisted intramolecular charge transfer (TICT) mechanism. However, the TICT tendency of dialkylated amino groups in biocompatible main‐stream fluorophores (i.e., coumarins and rhodamines) is weak, limiting their sensitivities. Herein, by replacing dialkylated amino donors with an *N*‐methylpyrrole group to enhance TICT, a simple and general method to engineer highly environmentally sensitive fluorescent probes is reported. This method yields a platter of colorful fluorescent probes that demonstrates outstanding polarity and viscosity sensitivity with large turn‐on ratios (up to 191 times for polarity and 14 times for viscosity), as well as distinct aggregation‐induced emission (AIE) characteristics. The utilities of these probes in both wash‐free bioimaging and protein detections are also successfully demonstrated. It is expected that this molecular design strategy will inspire the creation of many environmentally sensitive probes.

## Graphical abstract

Replacing a dialkylated amino donor with an *N*‐methylpyrrole group effectively enhances twisted intramolecular charge transfer (TICT) tendency in biocompatible main‐stream fluorophores. This simple and general method leads to a series of probes with greatly enhanced environmental sensitivity for biological applications and AIEgen development.

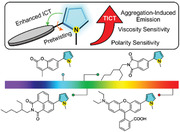

## 1 Introduction

Environmentally sensitive fluorescent probes are indispensable tools for studying biological systems with a high spatial and temporal resolution, vivid visibility, and excellent specificity.^[^ ^1^, ^2^ ^]^ Fluorescent output (i.e., intensities, wavelengths, or lifetime) of these probes experience substantial changes in response to analytes or environmental changes, thus allowing both qualitative and quantitative detections.^[^ ^3^, ^4^ ^]^ To this end, the twisted intramolecular charge transfer (TICT)^[^ ^5^, ^6^, ^7^, ^8^, ^9^ ^]^ mechanism has been extensively utilized in developing numerous fluorescent probes,^[^ ^9^ ^]^ such as viscosity sensors,^[^ ^10^, ^11^ ^]^ polarity probes,^[^ ^12^ ^]^ temperature sensors,^[^ ^13^ ^]^ and chemical probes.^[^ ^14^ ^]^ Yet, owing to their weak TICT tendencies, the environmental sensitivities of many biocompatible main‐stream fluorophores (such as coumarin and rhodamine derivatives) remain suboptimal. This poses a major limitation to advanced biological applications that demand high signal‐to‐noise ratios. It is thus imperative to formulate general design strategies that significantly boost the environmental sensitivities of these fluorophores, while maintaining their excellent biocompatibility.

Many existing TICT‐based fluorescent probes are push‐pull dyes employing dialkylated amino groups as the electron‐donating group (EDG).^[^ ^15^, ^16^, ^17^, ^18^, ^19^, ^20^ ^]^ Upon photoexcitation, the amino group could experience an intramolecular rotation of ≈90° with respect to the fluorophore scaffold, resulting in a perpendicular alignment in the excited state.^[^ ^7^, ^8^ ^]^ This rotation is accompanied by a complete charge separation between the EDG and the fluorophore scaffold. The TICT state is nonemissive and typically formed in polar solvents (which greatly stabilize the charged‐separated TICT species). Consequently, inhibiting TICT formations via environmental changes (i.e., via local viscosity variations, polarity changes, or other chemical reactions) recovers the fluorescence and prolongs fluorescence lifetime,^[^ ^21^ ^]^ thus allowing the assessment of the microenvironmental changes or target analytes.^[^ ^12^, ^22^ ^]^ However, the twisting ability of dialkylated groups is weak in many main‐stream fluorophores.

Notably, Rettig and co‐workers showed that pretwisting amino groups in the ground state facilitates TICT rotations in rhodamines, and the emission intensities of such dyes exhibited a much higher temperature sensitivity in comparison to other rhodamines with flat amino groups.^[^ ^23^ ^]^ Liu et al. showed that enhanced intramolecular charge transfer (ICT) led to considerable TICT in piperazine substituted naphthalimide, as the additional nitrogen atom increased the electron‐donating strength of the adjacent amino group via inductive effect.^[^ ^24^ ^]^ Yet, these design strategies employ bulky and hydrophobic substituents, potentially deteriorating the biocompatibility of the resulting probes.

**Figure 1:**
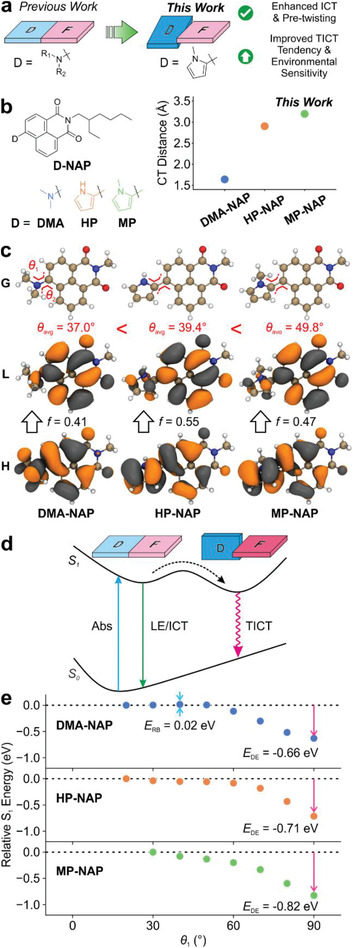
a) Illustration of molecular design strategy combining both ICT and pretwisting for enhancing TICT and environmental sensitivity. b) Molecular structures and the corresponding charge transfer distances in the Frank‐Condon state of DMA‐NAP, HP‐NAP, and MP‐NAP. c) S_0_ molecular geometries (G), HOMO (H), and LUMO (L) calculated at the M06‐2X/def2‐SVP level in toluene. The oscillator strength (*f*) of HOMO → LUMO transition is labeled in the inset. d) Illustration of S_0_ and S_1_ PESs for TICT formations. e) Calculated S_1_ PESs at the M06‐2X/def2‐SVP level in DMSO using cLR solvent formalism. The rotation barrier (*E*_RB_) and driving energy (*E*_DE_) for TICT are labeled in the inset.

Herein, by combining the enhancement of ICT and the enlargement of pre‐twisting, we reported *N*‐methylpyrrole substituted fluorophores that displayed substantially enhanced TICT tendency and greatly improved environmental sensitivity (i.e., to viscosity or polarity) in comparison to their dialkylated amino analogs (**Figure** **1**a). Our method is applicable to various chemical families of fluorophores, such as naphthalimide, phthalimide, coumarin, and rhodamine, yielding a platter of biocompatible dyes with notable aggregation‐induced emission (AIE) characteristics, sensitive protein detection capabilities, and high‐contrast live‐cell wash‐free bioimaging utilities.

## 2 Results and Discussion

### 2.1 Molecular Design and the Theoretical Validations

We hypothesized that replacing the dialkylated amino group with an *N*‐methylpyrrole group could greatly enhance the TICT tendency and improve the environmental sensitivities of the resulted fluorophores, for a few considerations (Figure 1a). First, *N*‐methylpyrrole (MP) has a stronger electron‐donating strength than dialkylated amino groups do, as suggested by the lower gas‐phase ionization potential (IP) of MP (7.94 eV)^[^ ^25^ ^]^ in comparison to that of dimethylamine (8.24 eV).^[^ ^26^ ^]^ Second, the steric hindrance of the methyl group in MP could enhance the pretwisting of this substituent with respect to the fluorophore scaffold, thus facilitating TICT rotations. Third, the MP group is small, and its substitution is likely to retain the biocompatibility of the parent fluorophore.

To verify our hypothesis, we designed three compounds in silico: dimethylamino (DMA‐NAP; the reference), pyrrole (HP‐NAP), and *N*‐methylpyrrole substituted naphthalimides (MP‐NAP) (Figure 1b, left panel). Between the latter two compounds, the addition of a methyl group on pyrrole not only increased steric hindrance, but also enhanced the electron‐donating strength of MP (IP = 7.94 eV)^[^ ^25^ ^]^ in comparison to that of HP (IP = 8.02 eV).^[^ ^27^ ^]^

We then conducted density‐functional theory (DFT) and time‐dependent density functional theory (TD‐DFT) calculations to compare the geometric and electronic structures of MP‐NAP and HP‐NAP against the reference compound DMA‐NAP. We noted several favorable features in MP‐NAP. First, in agreement with the increasing electron‐donating strength of DMA < HP < MP, calculated charge transfer (CT) distance (based on the centroids of the excited state density) progressively increases from DMA‐NAP (1.641 Å) to HP‐NAP (2.923 Å) and MP‐NAP (3.194 Å). These data suggest that ICT is significantly enhanced in MP‐NAP compared with DMA‐NAP and HP‐NAP (Figure 1b, right panel). Second, the pretwisting in MP‐NAP (*θ*_avg_ = 49.8°) is much more significant than that in DMA‐NAP (*θ*_avg_ = 37.0°) and HP‐NAP (*θ*_avg_ = 39.4°; Figure 1c).

Owing to the considerable ICT and significant pretwisting, calculations showed that MP‐NAP experiences a barrierless transition to the dark TICT state on S_1_ potential energy surface, while this rotation in DMA‐NAP is accompanied by a small energy barrier (0.02 eV; Figure 1d,e). Moreover, the driving force of TICT formation in MP‐NAP (−0.82 eV) is also much stronger than that of DMA‐NAP (−0.66 eV) and HP‐NAP (−0.71 eV). Hence, we expect that MP‐NAP should be completely dark in polar and nonviscous medium (due to effective TICT formations) and possess significant fluorescence turn‐on in low polarity and viscous media (when TICT formations are hindered) (Figures S1 and S2, Supporting Information).

Finally, it is worth mentioning that the pyrrole substituent extensively participates in the frontier molecular orbitals of MP‐NAP (especially in HOMO), and thus effectively expands the conjugation of naphthalimide. Owing to this expanded *π*‐conjugation, the oscillator strength of MP‐NAP (*f* = 0.52) is larger than that of DMA‐NAP (*f* = 0.44). This result suggests that the replacement of the dimethylamino group with the *N*‐methylpyrrole could also improve the molar absorption coefficients of naphthalimides. The molar extinction coefficient is an important parameter to improve the brightness of TICT probes when the fluorescence of the probes is on.

### 2.2 Experimental Validations of the Environmental Sensitivities of MP‐NAP

**Figure 2:**
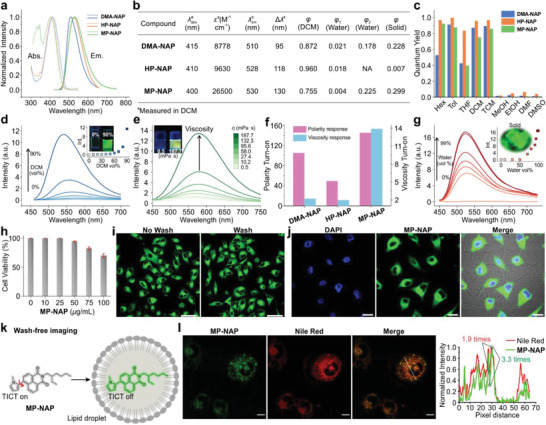
a) Normalized UV–vis and fluorescence spectra of DMA‐NAP, HP‐NAP, and MP‐NAP in DCM. b) The corresponding spectroscopic data measured in DCM and fluorescence quantum yields in water/MeOH mixture (water vol. 99%) and solid‐state. [dye] = 1.0 × 10^–5^ m for *φ*_1_ (monomers) and 3.5 × 10^–5^ for *φ*_2_ (aggregates), respectively. NA refers to not applicable as *φ* of these dyes are too small to detect. c) Quantum yields in various solvents. d) Fluorescence spectra of MP‐NAP in mixtures with varied DCM/MeOH volume ratios. The inset shows the peak fluorescence intensity as a function of the DCM volume fraction and the corresponding photographs. e) Fluorescence spectra of MP‐NAP in the mixture of MeOH and glycerol with varied viscosities; the inset shows the corresponding photographs. f) A comparison of polarity and viscosity turn‐on ratios of DMA‐NAP, HP‐NAP, and MP‐NAP. g) Fluorescence spectra of MP‐NAP in MeOH with different volume fractions of water. The inset shows the peak fluorescence intensity as a function of the water volume and the corresponding photograph in the solid state. h) Viability of cells after staining in MP‐NAP solutions of various concentrations for 4 h. Sample size = 16. i) Fluorescence images of A549 cells stained with MP‐NAP with and without 3 PBS washes. [dye] = 10 µg mL^−1^. j) Fluorescence images of A549 cells co‐stained with DAPI and MP‐NAP. [dye] = 10 µg mL^−1^. k) Schematic illustration of the wash‐free bioimaging of lipid droplets using MP‐NAP. l) Confocal microscopy of live HeLa cells co‐stained with MP‐NAP and Nile Red; the right panel shows the intensity profile along the white line highlighted in the left panel. [MP‐NAP] = 10 × 10^−6^ m; [Nile Red] = 1 × 10^−6^ m. Abbreviations of various solvents: Hex: hexane; Tol: toluene; THF: tetrahydrofuran; DCM: dichloromethane; TCM: trichloromethane; MeOH: methanol; EtOH: ethanol; DMF: dimethylformamide; DMSO: dimethyl sulfoxide. Scale bars: 50 µm for (i) and (j), 20 µm for (l).

Inspired by these theoretical predictions, we synthesized DMA‐NAP, HP‐NAP, and MP‐NAP and characterized their photophysical properties in a range of solvents with different polarities (**Figure** **2**a,b; Figures S3–S13; Schemes S1–S3, Supporting Information).^[^ ^16^, ^28^ ^]^ MP‐NAP emitted bright fluorescence in nonpolar solvents. The UV–vis absorption and fluorescence spectra of MP‐NAP peaked at 400 and 530 nm in dichloromethane, respectively (Figure 2a). The large Stokes shift of 130 nm is attributed to both strong intramolecular charge transfer and substantial geometry relaxations during photoexcitation.^[^ ^29^ ^]^ Notably, in dichloromethane, MP‐NAP possesses a high quantum yield of 0.755, along with a large maximum molar extinction coefficient of ≈26 500 M^–1^ cm^–1^. While the reference compounds DMA‐NAP and HP‐NAP also have high quantum yields (0.872 and 0.960) in dichloromethane, their peak molar extinction coefficients are 8778 and 9630 M^–1^ cm^–1^, respectively. Accordingly, the *N*‐methylpyrrole substitution leads to more than two folder increments in the overall brightness in a low polarity environment.

In contrast, the quantum yields of MP‐NAP in polar solvents (i.e., methanol, ethanol, and DMSO) are close to 0 (Figure 2c). To further demonstrate this polarity dependence, we measured the emission intensities of MP‐NAP in the mixture of DCM and methanol with varied volume ratios. When we increased the fraction of DCM to reduce solvent polarity, the emission intensities of MP‐NAP experienced a rapid enhancement (Figure 2d). This intensity‐polarity dependence is a typical feature of TICT.

To further confirm TICT rotations in polar solvents, we also measured the intensity‐viscosity dependence of MP‐NAP in the mixture of methanol and glycerol. As the viscosity increases, the emission intensity of MP‐NAP intensifies by >13 times, owing to the inhibition of TICT rotations in viscous media (Figure 2e).

For comparison, we also investigated the polarity and viscosity responses of DMA‐NAP and HP‐NAP under the same experimental conditions. The emission intensities of these two compounds increased by only ≈2 times as we varied solvent viscosities (Figure 2f, Figures S14–S17, Supporting Information), which is only about 1/6 of that in MP‐NAP. Accordingly, our experiments confirmed that MP‐NAP has a stronger TICT tendency in polar solvents and higher sensitivities toward viscosity changes. The contrast of its emission intensities from polar to nonpolar environments is also more substantial, due to its doubled maximum molar extinction coefficient in comparison to that of DMA‐NAP or HP‐NAP.

### 2.3 AIE Properties of MP‐NAP

In the last two decades, research on AIEgens has attracted significant research interests, owing to their broad applications (i.e., in bioimaging, biosensing, photodynamic therapy, and display technologies).^[^ ^30^, ^31^ ^]^ Encouraged by its outstanding environmental sensitivities, i.e., TICT‐induced quenching in polar solvents, we predicted that MP‐NAP could also show outstanding AIE behaviors as molecular aggregation could effectively reduce local polarities and hinder TICT rotations.^[^ ^9^, ^21^ ^]^ Moreover, the steric hindrance (with a pretwisted molecular structure) may also help to minimize intermolecular *π*‐*π* interactions for avoiding aggregation‐caused quenching (ACQ), thus endowing MP‐NAP with bright emissions in molecular aggregates and the solid state.

We thus measured the fluorescence spectra of MP‐NAP in the mixture of methanol (high solubility) and water (low solubility). As expected, the peak fluorescence intensity sharply increased when the volume fraction of water exceeds 75% (Figure 2g) as molecular aggregates were formed. The emission intensification is up to 160 times as the volume fraction of water increased from 0 to 99%. Subsequent measurements showed that the solid powder of MP‐NAP showed an absolute quantum yield of 0.299 (Figure 2b), corroborating the excellent AIE properties of MP‐NAP. In comparison, though DMA‐NAP showed similar AIE properties, its emission intensification was less than 30 times upon adding water into the methanol solution (Figure S18, Supporting Information). These data again demonstrated the outstanding environmental sensitivity of *N*‐methylpyrrole substituted naphthalimide.

### 2.4 Live‐Cell Wash‐Free Bioimaging Using MP‐NAP

Subsequently, we explored the wash‐free bioimaging utilities of MP‐NAP in live cells. In conventional bioimaging experiments, a wash step is routinely required to remove unbounded or weakly bounds dyes to minimize background interference. This wash step wasted imaging contrast agents and could induce significant cell damages to delicate samples. In contrast, wash‐free dyes effectively circumvented these disadvantages by quenching the background fluorescence of unbounded dyes. Given that MP‐NAP is nonemissive in polar solvents, this probe (of low concentrations) should remain dark in aqueous cellular media but turn on bright emissions when binding with nonpolar biomolecules. We thus speculated that MP‐NAP could be used in wash‐free bioimaging of live cells.

We next verified this hypothesis with live A549 cell lines. We first investigated the cytotoxicity of MP‐NAP. Our experiments showed that the cell viability was ≈100% when the concentration of MP‐NAP was in the range of 10–50 µg mL^−1^ (Figure 2h). Further cell viability tests revealed that >70% of cells remained alive after 4 h even at a high concentration of MP‐NAP (100 µg mL^−1^). These measurements showed that MP‐NAP has low cytotoxicity. Moreover, we noted that MP‐NAP could easily penetrate cell membranes.

We thus performed bioimaging experiments with and without the wash step to compare the imaging quality of MP‐NAP (Figure 2i). Our results showed that cells without the wash step displayed high contrast and bright green fluorescence, similar to those with the wash step. These data indicate that MP‐NAP could be employed as a wash‐free fluorescent dye. We also compared the performance of DMA‐NAP, HP‐NAP, and MP‐NAP on wash‐free imaging. The results suggested that MP‐NAP performed the best as it provided clear outlines of the cells (Figure S19, Supporting Information). Moreover, our data revealed that MP‐NAP stayed in the cytoplasm but could not enter the nucleus. Using both MP‐NAP and DAPI (4′, 6‐diamidino‐2‐phenylindole; blue stains of the nucleus), we successfully performed multi‐colored fluorescence imaging of live A549 cells (Figure 2j).

Inspired by these imaging results, we envisioned that MP‐NAP could preferentially stain lipid droplets (LDs). LDs are lipid‐rich cellular organelles that play a crucial role in maintaining lipid bilayers and storing energy.^[^ ^32^ ^.^ ^33^ ^]^ LDs are highly viscous (≈50 cP) and provide a nonpolar environment,^[^ ^34^ ^]^ which inhibits TICT. Notably, MP‐NAP possesses strong lipophilicity. This high lipophilicity of MP‐NAP is reflected by the calculated log *P* (Clog*P*) value of 7.43, while Clog*P*>3 suggests strong lipophilicity.^[^ ^35^ ^]^ MP‐NAP could thus enter the viscous and nonpolar LDs, emitting bright fluorescence (due to the suppression of TICT). Meanwhile, owing to the accumulation of MP‐NAP in LDs, the potential formation of MP‐NAP aggregates could also trigger AIE, lighting up LDs (Figure 2k).

Indeed, our subsequent imaging experiments using confocal microscopy showed that the fluorescence signals of MP‐NAP overlapped very well with those of Nile Red (a commercially available lipid droplet marker) in live HeLa cells (Figure 2l). Moreover, the fluorescence intensity fluctuations along a line showed that MP‐NAP afforded a higher signal‐to‐noise ratio than Nile Red. Additional experiments also showed that MP‐NAP outperformed DMA‐NAP and HP‐NAP with an approximately doubled Pearson's coefficient (Figures S20 and S21; Table S1, Supporting Information). These results demonstrate that MP‐NAP is a good candidate for wash‐free imaging of lipid droplets.

### 2.5 Generalization of the *N*‐Methylpyrrole Substitution Strategy

Inspired by the excellent environmental sensitivity of MP‐NAP and its (wash‐free) bioimaging utilities, we next explored the generalizability of our *N*‐methylpyrrole substitution strategy in other chemical families of fluorophores, such as phthalimide (PHA), coumarin (COU), and rhodamine (RHO). It is of note that PHA and COU were often recognized as the ICT fluorophores, while the photoexcitation of RHO leads to a locally excited (LE) state.

**Figure 3:**
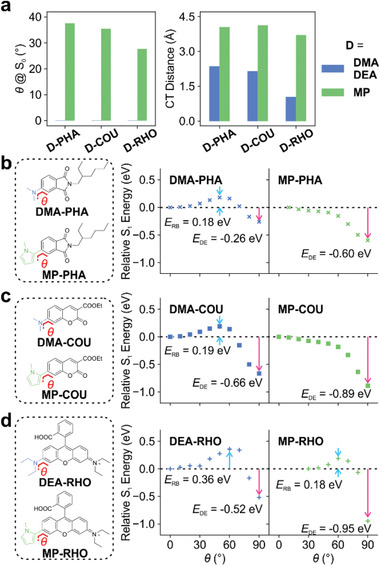
a) The pre‐twisting angles based on the S_0_ geometries and CT distances of FC states in various dialkylated amino and *N*‐methylpyrrole substituted fluorophores. The comparison of S_1_ PESs for TICT formations in b) DMA‐PHA versus MP‐PHA, c) DMA‐COU versus MP‐COU, and d) DEA‐RHO versus MP‐RHO.

We first compared their molecular geometries in terms of the pretwisting of the electron‐donating groups, and the degree of ICT in terms of the CT distances (**Figure** **3**a, Figure S22, Supporting Information). Our calculations showed that the *N*‐methylpyrrole substitution results in the substantially enlarged pretwisting ranging from 27.7° to 37.6° compared with their dialkylamino analogs (≈0.0°).

Meanwhile, the replacement of these dialkylamino groups with *N*‐methylpyrrole greatly enhanced ICT, as the CT distances were approximately doubled in PHA (from 1.641 to 3.194 Å) and COU (from 2.152 to 4.123 Å) and tripled in RHO (from 1.051 to 3.706 Å).

Owing to the enhancement of both pre‐twisting and ICT, our subsequent S_1_ potential energy surface calculations showed that MP‐PHA, MP‐COU, and MP‐RHO demonstrated lower energy barriers and stronger driving energy to enter the TICT states, in comparison to their corresponding dialkylamino analogs (Figure 3b–d, Figures S23–S26, Supporting Information). These results indicate that our method is generalizable to both ICT and LE fluorophores for enhancing TICT formations.

### 2.6 Validation of the *N*‐Methylpyrrole Substitution Strategy in Various Fluorophores

These computational results motivated us to synthesize MP‐PHA, MP‐COU, MP‐RHO, and characterize their photophysical properties (**Figure** **4**a, Schemes S4–S6 and Figures S27–S43, Supporting Information). Similar to MP‐NAP, MP‐PHA and MP‐COU displayed high quantum yields (0.6–1.0) in low‐polarity solvents but became nonemissive (quantum yields ≈0.01) in polar and/or protonic solvents (Figure 4b). Notably, newly synthesized MP‐PHA exhibited a more than twofold increase in *ε* (21 400 M^–1^ cm^–1^)^[^ ^16^, ^36^ ^]^ in comparison to conventional PHA derivatives. Besides, MP‐COU showed a favorable bathochromic shift (>40 nm) compared with its dialkylamino analogs.^[^ ^17^ ^]^

**Figure 4:**
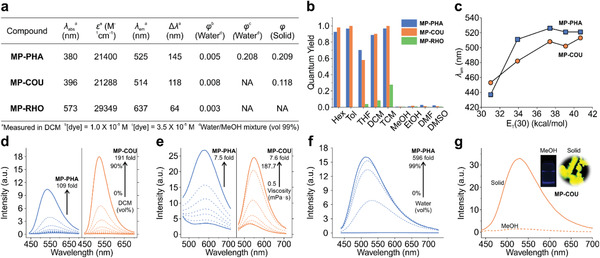
a) Spectroscopic data of MP‐PHA, MP‐COU, and MP‐RHO in DCM and fluorescence quantum yields in water and solid‐state. NA refers to not applicable as *φ* of these dyes are too small to detect. b) Quantum yields in various solvents. c) Emission wavelength as a function of solvent polarity scale, *E*_T_(30). d) Fluorescence spectra of MP‐PHA and MP‐COU in mixtures with varied DCM/MeOH volume ratios. e) Fluorescence spectra of MP‐PHA and MP‐COU in the mixtures of MeOH and glycerol with varying viscosities. f) Fluorescence spectra of MP‐PHA in solutions with varied MeOH/water volume ratios. g) Fluorescence spectra of MP‐COU in solid‐state and MeOH. The inset shows the corresponding photographs.

MP‐PHA and MP‐COU also experienced enhanced fluorescence contrast going from nonpolar to polar environments or from low to high viscosity media (Figure 4c–e). To characterize their AIE properties, we noted that the fluorescence intensity of MP‐PHA displayed an enhancement of ≈596 times when the solvent was changed from pure methanol to methanol/water ‐mixture (1:99 in volume; Figure 4f). This enhancement is attributed to the formation of molecular aggregates. Similarly, MP‐COU was highly fluorescent in the solid state (*φ* = 0.118), in contrast to the negligible emissions in methanol as monomers (*φ* = 0.007; Figure 4g).

**Figure 5:**
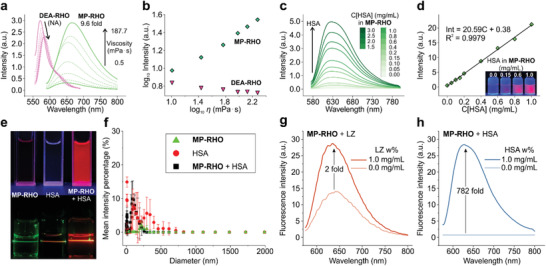
a) Fluorescence response of MP‐RHO and DEA‐RHO in the mixtures of MeOH and glycerol with varying viscosities. b) Fluorescence intensities of MP‐RHO and DEA‐RHO as a function of viscosity in logarithmic scales. c) Fluorescence spectra of MP‐RHO in urine upon the addition of human serum albumin. d) The correlation between fluorescence intensity and the concentration of human serum albumin in (c). The inset photographs show the change of fluorescence of MP‐RHO in urine with varied concentrations of human serum albumin. e) Images of MP‐RHO, HSA, and MP‐RHO + HSA solution under UV irradiations (top panel) and a green laser (bottom panel). f) DLS spectra of MP‐RHO, HSA, and MP‐RHO + HSA solution. Comparison between the fluorescence responses of MP‐RHO to g) LZ and h) HSA.

Interestingly, the MP‐RHO solution was colorless and nonfluorescent, indicating the formation of the closed form of lactones.^[^ ^37^, ^38^ ^]^ For this reason, the photophysical behavior of MP‐RHO was studied in the presence of 0.1% trifluoroacetic acid. While the absolute quantum yields of MP‐RHO are low in general, quantum yields of MP‐RHO are still relatively higher in nonpolar solvents than in polar solvents (Figure 4b, Figure S44, Supporting Information). For example, the quantum yield of MP‐RHO in TMC (0.277) is ≈30 times as large as those in polar solvents (<0.01). In comparison to the parent compound DEA‐RHO, the replacement of *N*,*N*‐diethylamino with *N*‐methylpyrrole substitution not only resulted in a bathochromic shift of ≈60 nm and a twofold increase in the Stokes shift (Figure 4a), but also endowed MP‐RHO with superior fluorescence response to viscosity (**Figure** **5**a, b). For instance, the fluorescence emission of MP‐RHO increased by ≈10 times as solvent viscosity rises from 0.5 to 187.7 cP (Figure 5a). In stark contrast, the fluorescence of DEA‐RHO decreased along with a slight redshift when the viscosity of the solution increased (Figure 5a and Figure S45, Supporting Information).

We also measured the photostability of MP‐NAP, MP‐PHA, MP‐COU, and MP‐RHO under continuous illuminations in DCM. These samples were excited at their respective peak UV–vis absorption wavelengths (Figure S46, Supporting Information). All these *N*‐methylpyrrole substituted fluorophores showed favorable photostability with an intensity drop of less than 2%. The good photostability of these dyes warrants further applications.

### 2.7 Albuminuria Detection in Artificial Urine by MP‐CHO

Currently, ≈10% of the world's population suffers from chronic kidney diseases. In patients with kidney diseases, albumin proteins from blood plasma could leach into the urine (namely albuminuria).^[^ ^39^ ^]^ Accordingly, the measurement of urine albumin is an important method for both diagnosing kidney diseases and monitoring their progression. To this end, a 24‐hour urine protein test is routinely required to quantify albumin, and the total amount of albumin found in urine has a maximum of 150 mg per day for a healthy person. This threshold translates to an albumin concentration of ≈0.10 mg mL^−1^ in urine.

It has been reported that serum albumin possesses a hydrophobic region, which serves as a docking site for a wide variety of substances, such as hormones and drugs.^[^ ^40^, ^41^ ^]^ Encouraged by the outstanding environmental sensitivity of MP‐RHO, we hypothesized that MP‐RHO may bind with albumin in an aqueous solution and produce fluorescence turn‐on, owing to the decrease in local polarity and the increase in viscosity for suppressing TICT.

Our subsequent experiments confirmed that MP‐RHO showed excellent performance in detecting bovine serum albumin (BSA) and human serum albumin (HSA) in PBS buffer (pH = 7.4; Figure 5c) and urine (pH = 4.7; Figures S47 and S48, Supporting Information). The varied pH values of these two samples were chosen to mimic different pH values in real urine samples. These tests led to similar results: upon the addition of HSA, the fluorescence intensity of MP‐RHO gradually increased and demonstrated a good linear relationship with the concentration of HSA (0–1 mg mL^−1^; Figure 5d). The calculated detection limit is 0.037 mg mL^−1^, which is much lower than the current requirement of the health standard (≈0.10 mg mL^−1^). Moreover, the detection can be easily visualized even with naked eyes (inset photo in Figure 5d). In comparison, the fluorescence of DEA‐RHO experienced only minor changes with the addition of HSA (Figure S48, Supporting Information).

We have verified that the detection mechanism of HSA is due to monomer binding, instead of molecular aggregation. To this end, we studied the degree of dissolution in MP‐RHO/protein systems based on the Tyndall effect and dynamic light scattering (DLS) experiments. We found that none of the samples (MP‐RHO, HSA, MP‐RHO/HSA) showed an obvious Tyndall effect (Figure 5e). Such observations suggested no significant formations of molecular aggregates, and MP‐RHO/HSA were well separated in solution. In DLS spectra, HSA showed two peaks around 120 and 300 nm (Figure 5f). MP‐RHO exhibited a vanishing peak at around 250 nm. A single peak around 120 nm was found in MP‐RHO/HSA. These results indicated that HSA provided hydrophobic pockets for MP‐RHO to enter. MP‐RHO was well separated in the solution and did not aggregate (as the 250 nm peak disappeared). The 300 nm peak in HSA also disappeared when MP‐RHO was added (in MP‐RHO/HSA), suggesting that the hydrophobic interaction between HSA and MP‐RHO occurred and this interaction resulted in a more compact configuration of HSA. These experiments ruled out the aggregation of MP‐RHO in the solution and confirmed the binding of HSA with MP‐RHO.

To further correlate the fluorescence turn‐on to TICT suppression induced by the hydrophobic interaction, we compared the fluorescence response of MP‐RHO in different proteins, i.e., in lysozyme (LZ) and HSA. The surface of LZ is relatively hydrophilic, while HSA has hydrophobic pockets. Therefore, LZ is expected to have a poor combination with MP‐RHO compared with HSA, leading to a reduced fluorescence turn‐on as TICT formation is still significant. Indeed, when we increased the concentration of lysozyme from 0.0 to 1.0 mg mL^−1^, the fluorescence intensity only slightly increased (Figure 5g). In HSA, the same amount of HSA induced a 782‐fold fluorescence turn‐on (Figure 5h). Overall, our results demonstrated that the fluorescence turn‐on in MP‐RHO is aroused from its interactions with HSA to suppress TICT formation.

These results collectively demonstrate the superior sensing performance of MP‐RHO in albuminuria measurement and proved the generalizability of the *N*‐methylpyrrole substitution strategy in producing highly environmentally sensitive fluorescent probes.

## 3 Conclusion

In summary, we reported a general *N*‐methylpyrrole substitution strategy for developing environmentally sensitive and biocompatible fluorescent probes. Owing to both the enhancement of intramolecular charge transfer and the enlargement of pretwisting, *N*‐methylpyrrole substituted fluorophores exhibited a strong tendency for TICT formations and negligible fluorescence in polar and nonviscous environments. Reducing solvent polarity and/or increasing viscosity significantly boost the emissions of these probes, thus endowing them outstanding environmental sensitivity. These probes also exhibit favorable aggregation‐induced‐emission characteristics. Finally, we have successfully demonstrated the bioimaging and biosensing utilities of these probes, in high contrast wash‐free live‐cell imaging and human serum albumin detection. We expect that our work will inspire the creation of highly environmentally sensitive TICT fluorophores with improved contrast for advanced biological applications.

## 4 Experimental Section

### Materials

All reagents were purchased from commercial suppliers and used without further purification. All the solvents used were at least analytical grade and obtained from China Sinopharm Chemical Reagent Co., Ltd. Water used for measurement was purified by the Millipore filtration system.

### Instrumentation

^1^H NMR and ^13^C NMR spectra were obtained on a Bruker AV 600 NMR spectrometer. High‐resolution mass spectra (HRMS) were obtained from the liquid chromatography coupled time‐of‐flight mass spectrometer with electrospray ionization (ESI), MAXIS, Brooker. UV–vis measurements were carried out on a U‐3900 (Hitachi) spectrophotometer. Fluorescence measurements were done on an FLS 920, Edinburgh. Absolute fluorescence quantum yields were recorded on a quantum efficiency measurement system (Hamamatsu, Quantaurus‐QY). Fluorescence images were taken on a laser scanning confocal microscopy (Leica, TCSSP8 for A549 cells; Olympus, FV1000 for HeLa cells).

### Cellular Uptake

A549 cell lines maintained in DMEM (Dulbecco’ Modified Eagle Medium) were used for all the cellular studies (cell viability and cellular uptake). Cells were treated with 10 µg mL^−1^ of **MP‐NAP** (in DMEM) for 4 h at 37 °C with 5% CO_2_. Afterward, the cells were used for cell viability tests or imaging. For multicolor imaging, cells were treated with **MP‐NAP** and **DAPI** (4′, 6‐diamidino‐2‐phenylindole) for 4 h at 37 °C 5% CO_2_. The cells were washed three times with PBS buffer before imaging. HeLa cells were cultured in DMEM medium supplemented with 10% fetal bovine serum (FBS), penicillin (100 units mL^−1^), streptomycin (100 units mL^−1^) at 37 °C in a humidified atmosphere of 5% CO_2_. To prepare for fluorescence imaging, HeLa cells were seeded in a 35 mm Petri dish with a coverslip at 37 °C overnight. The cells were treated with probes ([DAM‐NAP] = 10 × 10^−6^ m or [HP‐NAP] = 10 × 10^−6^ m or [MP‐NAP] = 10 × 10^−6^ m) for 30 min. The cells were then washed twice with PBS. Subsequently, the medium was replaced with a fresh medium containing 1 × 10^−6^ m of Nile Red and incubated for another 15 min at 37 °C. After washing the cells with PBS three times, the fluorescence images were taken.

### Computational Details

All quantum chemistry calculations were performed with Gaussian 16 software.^[^ ^42^ ^]^ The molecular geometries and the corresponding properties were calculated at the M062X/def2‐SVP level in SMD solvents. M062X functional combined with the corrected linear response solvent formalism^[^ ^43^ ^]^ (M062X/cLR‐SMD) was used for describing S_1_ potential energy surface (PES) for TICT formations in DMSO.^[^ ^15^ ^]^ The charge transfer distance (*d*_CT_)^[^ ^44^ ^]^ was calculated using Multiwfn 3.3.6.^[^ ^45^ ^]^

### Statistical Analysis

The absorption and emission spectra in Figure 2a were normalized. All the fluorescence intensities labeled in a.u. were divided by 1000 with respect to the original data. The sample size in Figure 2h was 16 and the corresponding SD was < 0.091. The colocalization coefficient was analyzed by the ImageJ software.

## Conflict of Interest

The authors declare no conflict of interest.

## Supporting information

Supporting Information

## Data Availability

The data that support the findings of this study are available in the supplementary material of this article.
