# Supplementary material for: A General Method to Develop Highly Environmentally Sensitive Fluorescent Probes and AIEgens
Source: Adv Sci (Weinh). 2021 Dec 19;9(5):2104609. doi: 10.1002/advs.202104609 (PMC8844555; doi:10.1002/advs.202104609)
Supplement: Supplementary file 1 — Supporting Information [file ADVS-9-2104609-s001.pdf]

ADVANCED SCIENCE
Open Access

Supporting Information

for *Adv. Sci.*, DOI: 10.1002/advs.202104609

A General Method to Develop Highly Environmentally Sensitive Fluorescent Probes and AIEgens

Rong Miao, Jing Li, Chao Wang, Xuefeng Jiang, Ying Gao, Xiaoling Liu, Dan Wang, Xin Li, Xiaogang Liu and Yu Fang**

Supporting Information

A General Method to Develop Highly Environmentally Sensitive Fluorescent Probes and AIEgens

Rong Miao, Jing Li, Chao Wang, Xuefeng Jiang, Ying Gao, Xiaoling Liu, Dan Wang, Xin Li, Xiaogang Liu and Yu Fang**

Table of Contents

1. Calculations of DMA-NAP, HP-NAP, and MP-NAP S6

Figure S1. Oscillator strength of DMA-NAP, HP-NAP, and MP-NAP as a function of rotation angle (θ) on the S_1 PES for TICT calculated at the M062X/def2-SVP/cLR-SMD level in DMSO........ S6

Figure S2. Molecular geometry and the distribution of HOMO and LUMO of DMA-NAP, HP-NAP, and MP-NAP at the LE/ICT and TICT states calculated at the M062X/def2-SVP/cLR-SMD level in DMSO. S7

2. Synthesis of DMA-NAP, HP-NAP, and MP-NAP S8

2.1 Synthesis of MP-NAP S8

Scheme S1. Synthesis route for MP-NAP. S8

Figure S3. ^{1}H NMR spectra of Br-NAP. S9

Figure S4. ^{13}C NMR spectra of Br-NAP S9

Figure S5. ^{1}H NMR spectra of MP-NAP S10

Figure S6. ^{13}C NMR spectra of MP-NAP S10

2.2 Synthesis of DMA-NAP S11

Scheme S2. Synthesis route for DMA-NAP. S11

Figure S7. ^{1}H NMR spectra of DMA-NAP. S11

Figure S8. ^{13}C NMR spectra of DMA-NAP. S12

2.3 Synthesis of HP-NAP S12

Scheme S3. Synthesis route for HP-NAP S12

Figure S9. ^{1}H NMR spectra of HP-NAP. S13

Figure S10. ^{13}C NMR spectra of HP-NAP. S13

3. Spectroscopic and bioimaging studies of DMA-NAP, HP-NAP, and MP-NAP S14

Figure S11. Fluorescence emission spectra of MP-NAP in different solvents. Concentration: 2.5×10^{-5} M. Hex: hexane; Tol: toluene; THF: tetrahydrofuran; DCM: dichloromethane; TCM: trichloromethane; MeOH: methanol; EtOH: ethanol; DMF: dimethylformamide; DMSO: dimethylsulfoxide. λ_{ex} = 400 nm. S14

Figure S12. Normalized fluorescence excitation (black) and emission (red) spectra of DMA-NAP in dichloromethane. Concentration = 3.0×10^{-5} M; λ_{ex} = 415 nm; λ_{em} = 510 nm. S14

Figure S13. Normalized fluorescence excitation (black) and emission (red) spectra of HP-NAP in dichloromethane. Concentration = 2.5×10^{-5} M; λ_{ex} = 410 nm; λ_{em} = 528 nm. S15

Figure S14. Fluorescence emission spectra of DMA-NAP in the mixtures of DCM and MeOH with varied volume fractions of DCM. DCM: dichloromethane; MeOH: methanol. Concentration = 3.0×10^{-5} M; λ_{ex} = 415 nm. S15

Figure S15. Fluorescence emission spectra of HP-NAP in the mixtures of DCM and MeOH with varied volume fractions of DCM. DCM: dichloromethane; MeOH: methanol. Concentration = 2.5×10^{-5} M; λ_{ex} = 410 nm. S16

Figure S16. Fluorescence emission spectra of DMA-NAP in the mixtures of methanol and glycerol with different viscosities. Concentration = 3.0×10^{-5} M; λ_{ex} = 415 nm...... S16

Figure S17. Fluorescence emission spectra of HP-NAP in the mixtures of methanol and glycerol with different viscosities. Concentration = 2.5×10^{-5} M; λ_{ex} = 410 nm. S17

Figure S18. Fluorescence emission spectra of DMA-NAP in the mixtures of H_2O and MeOH with varied volume fractions of water. Concentration = 3.0×10^{-5} M; λ_{ex} = 415 nm. S17

Figure S19. Fluorescence imaging of control cells (without dyes) and live cells treated with MP-NAP, HP-NAP, and DMA-NAP. Dye concentration: 10 µg/mL; incubation time: 4 hours at 37 °C; 5% CO_2.. S17

4. Calculations of DMA-PHA, MP-PHA, DMA-COU, MP-COU, DEA-RHO, and MP-RHO.. S20

Figure S20. Molecular geometry and the distribution of HOMO and LUMO of (a) DMA-PHA and MP-PHA, (b) DMA-COU and MP-COU, and (c) DEA-RHO and MP-RHO at the FC state calculated at the M062X/def2-SVP level in toluene. The oscillator strength and CT distance are labelled in the inset. S20

Figure S21. Oscillator strength of (a) DMA-PHA and MP-PHA, (b) DMA-COU and MP-COU, and (c) DEA-RHO and MP-RHO as a function of rotation angle (θ) on the S_1 PES for TICT calculated at the M062X/def2-SVP/cLR-SMD level in DMSO. S20

Figure S22. Molecular geometry and the distribution of HOMO and LUMO of DMA-PMD and MP-PMD at the (a) LE/ICT and (b) TICT states calculated at the M062X/def2-SVP/cLR-SMD level in DMSO. S21

Figure S23. Molecular geometry and the distribution of HOMO and LUMO of DMA-COU and MP-COU at the (a) LE/ICT and (b) TICT states calculated at the M062X/def2-SVP/cLR-SMD level in DMSO. S21

Figure S24. Molecular geometry and the distribution of HOMO and LUMO of DEA-RHO and MP-COU at the (a) LE/ICT and (b) TICT states calculated at the M062X/def2-SVP/cLR-SMD level in DMSO. S22

5. Synthesis of MP-PHA, MP-COU, and MP-RHO S23

5.1 Synthesis of MP-PHA.. S23

Scheme S4. Synthesis route for MP-PHA.. S23

Figure S25. ^{1}H NMR spectra of Br-PHA. S23

Figure S26. ^{13}C NMR spectra of Br-PHA. S24

Figure S27. ^{1}H NMR spectra of MP-PHA. S24

Figure S28. ^{13}C NMR spectra of MP-PHA. S25

5.2 Synthesis of MP-COU S25

Scheme S5. Synthesis route for MP-COU S25

Figure S29. ^{1}H NMR spectra of MP-OH. S26

Figure S30. ^{13}C NMR spectra of MP-OH. S27

Figure S31. ^{1}H NMR spectra of MP-COU. S27

Figure S32. ^{13}C NMR spectra of MP-COU. S28

5.3 Synthesis of MP-RHO S28

Scheme S6. Synthesis route for MP-RHO S28

Figure S33. ^{1}H NMR spectra of I-RHO. S29

Figure S34. ^{13}C NMR spectra of I-RHO. S30

Figure S35. ^{1}H NMR spectra of MP-RHO. S30

Figure S36. ^{13}C NMR spectra of MP-RHO. S31

6. Spectroscopic study of DMA-NAP, HP-NAP, and MP-NAP S32

Figure S37. Fluorescence emission spectra of MP-PHA in different solvents. Hex: hexane; Tol: toluene; THF: tetrahydrofuran; DCM: dichloromethane; TCM: trichloromethane; MeOH: methanol; EtOH: ethanol; DMF: dimethylformamide; DMSO: dimethylsulfoxide. Concentration = 2.5×10^{-5} M. λ_{ex} = 375 nm. S32

Figure S38. Fluorescence emission spectra of MP-COU in different solvents. Hex: hexane; Tol: toluene; THF: tetrahydrofuran; DCM: dichloromethane; TCM: trichloromethane; MeOH: methanol; EtOH: ethanol; DMF: dimethylformamide; DMSO: dimethylsulfoxide. Concentration = 2.0×10^{-5} M; λ_{ex} = 400 nm. S32

Figure S39. Normalized fluorescence excitation (black) and emission (red) spectra of MP-PHA in dichloromethane. Concentration = 2.5×10^{-5} M; λ_{ex} = 375 nm; λ_{em} = 525 nm. S33

Figure S40. Normalized fluorescence excitation (black) and emission (red) spectra of MP-COU in dichloromethane. Concentration = 2.0×10^{-5} M; λ_{ex} = 400 nm; λ_{em} = 510 nm. S33

Figure S41. Fluorescence emission spectra of MP-RHO in different solvents. DCM: dichloromethane; TCM: trichloromethane; MeOH: methanol; EtOH: ethanol; DMSO: dimethylsulfoxide. Concentration = 1.7×10^{-5} M; λ_{ex} = 575 nm. S33

Figure S42. Fluorescence excitation (green) and emission (red) spectra of DEA-RHO (dashed lines) and MP-RHO (solid lines) in dichloromethane. Concentrations of the compounds = 2.0×10^{-5} M. All measurements for MP-RHO were conducted in the presence of 0.1% (*v*/*v*) trifluoroacetic acid. DEA-RHO: λ_{ex} = 550 nm; λ_{em} = 570 nm. MP-RHO: λ_{ex} = 575 nm; λ_{em} = 630 nm. S34

Figure S43. Fluorescence emission spectra of DEA-RHO in the mixtures of methanol and glycerol with different viscosities. Concentration = 2.0×10^{-5} M; λ_{ex} = 550 nm...... S34

7. Photostability of *N*-methylpyrrole substituted fluorophores........ S35

Figure S44. Photostability of the N-methylpyrrole probes. (a) Plots of fluorescence intensity of the fluorophores *vs* continuous irradiation time at the maximum excitation wavelength of each fluorophore in dichloromethane. (b) Calculated photobleaching efficiency for each fluorophore. Irradiation wavelength/monitoring wavelength: MP-NAP (400/530 nm); MP-PHA: (375/525 nm); MP-COU: (400/510 nm); MP-RHO: (575/630 nm). Concentration of the probes = 2.5×10^{-5} M........ S35

8. Albuminuria detection in artificial urine S36

Figure S45. Fluorescence emission spectra of MP-RHO in PBS with different concentrations of BSA (a) and HSA (b). The measurements were conducted in the presence of 0.1% (*v*/*v*) trifluoroacetic acid. Concentration of MP-RHO = 1.8×10^{-5} M; λ_{ex} = 575 nm........ S36

Figure S46. Fluorescence emission spectra of DEA-RHO in urine (pH = 4.7) with different amounts of HSA. Concentration = 1.3×10^{-5} M; λ_{ex} = 550 nm. S36

9. References S37

1. Calculations of DMA-NAP, HP-NAP, and MP-NAP

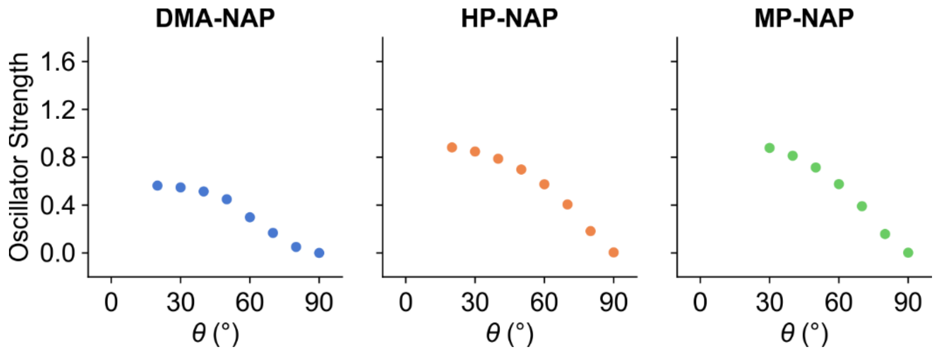

Figure S1. Oscillator strength of DMA-NAP, HP-NAP, and MP-NAP as a function of rotation angle (θ) on the S_1 PES for TICT calculated at the M062X/def2-SVP/cLR-SMD level in DMSO.

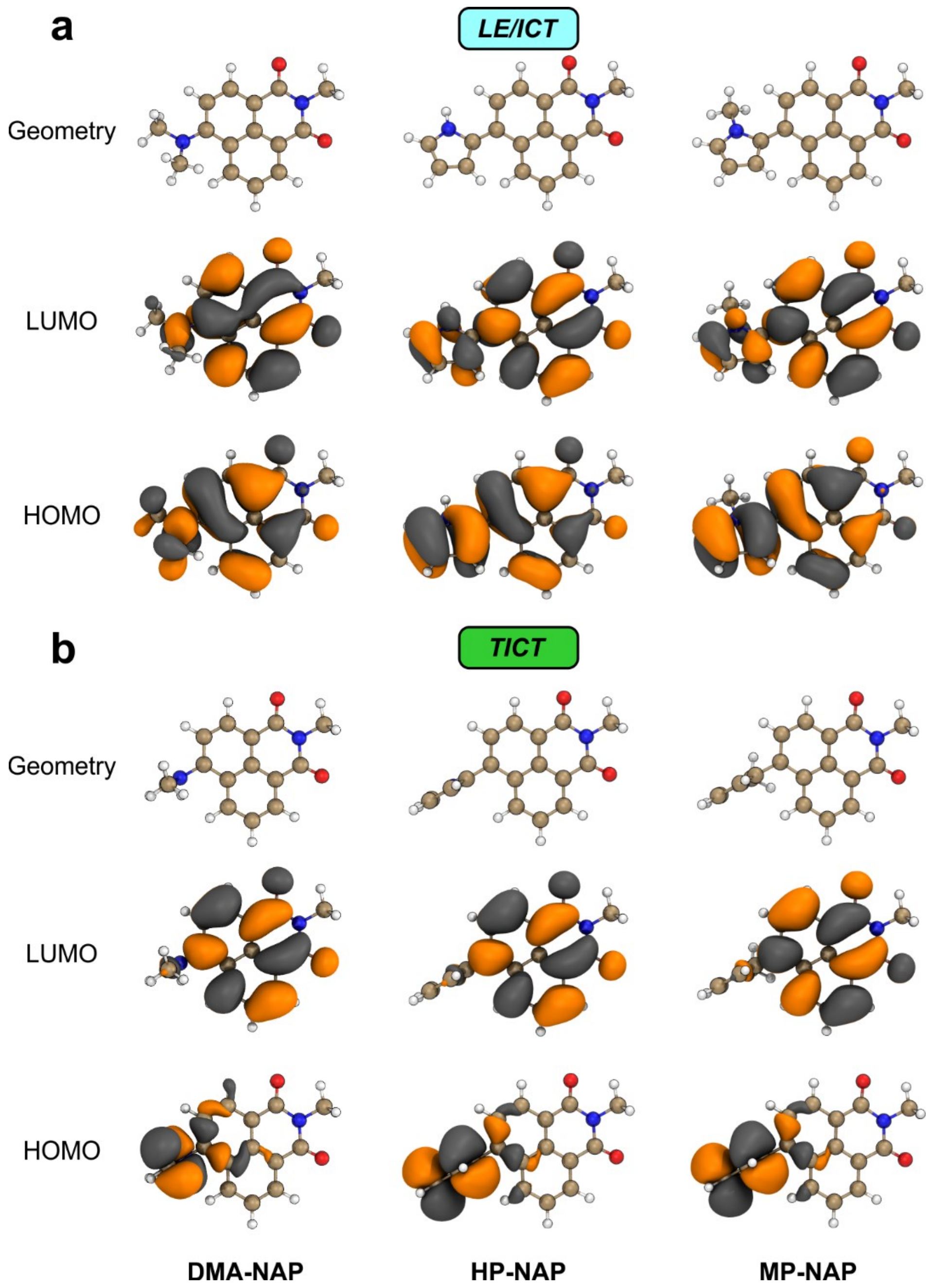

Figure S2. Molecular geometry and the distribution of HOMO and LUMO of DMA-NAP, HP-NAP, and MP-NAP at the LE/ICT and TICT states calculated at the M062X/def2-SVP/cLR-SMD level in DMSO.

2. Synthesis of DMA-NAP, HP-NAP, and MP-NAP

2.1 Synthesis of MP-NAP

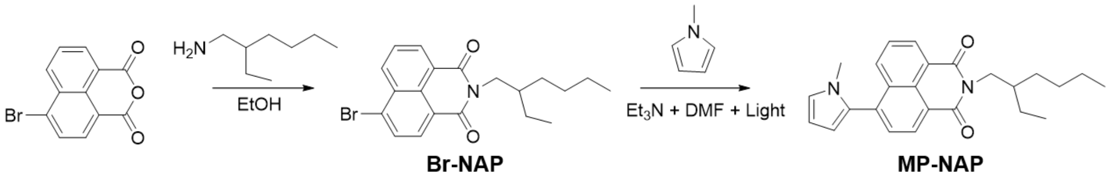

Scheme S1. Synthesis route for MP-NAP.

1.35 g of 4-Bromo-1, 8-naphthalic anhydride (5 mmol) was dissolved in 35 mL glacial acetic acid. 0.65 mL of 2-ethylhexylamine was added to the solution and then the mixture was heated to reflux. After 6 hours, the mixture was cooled down. The precipitate was filtrated and repeatedly washed with ethanol and water. 720 mg of Br-NAP was obtained as light yellow solids. 234 mg (0.6 mmol).

MP-NAP was synthesized according to our reported work.[1] Br-NAP was dissolved in 12 mL dimethylformamide in a 50 mL vial with a magnetic stirring bar. The solution was degassed by N_2 via a syringe needle for 30 min, and 180 μL Et_3N was added during the degassing process. 1.8 mL of N-methyl pyrrole was added into the mixture when the degassing is completed. Afterwards, the vial was sealed up and the mixture was irradiated through the bottom of the vial using LED light. The reaction was monitored through a thin-layer chromatography (TLC) analysis. When the reaction is completed, the reaction mixture was transferred into a 50 mL round-bottom flask and then concentrated in vacuum. Purification of the crude product was achieved by column chromatography using petrol ether/ethyl acetate (10:1) on a silica gel column. MP-NAP was obtained as yellow-green solids with a yield of 68%. HRMS calculated for $[C_{13}H_{13}NO_2Na]^+$: 411.2048; found: 411.2045.

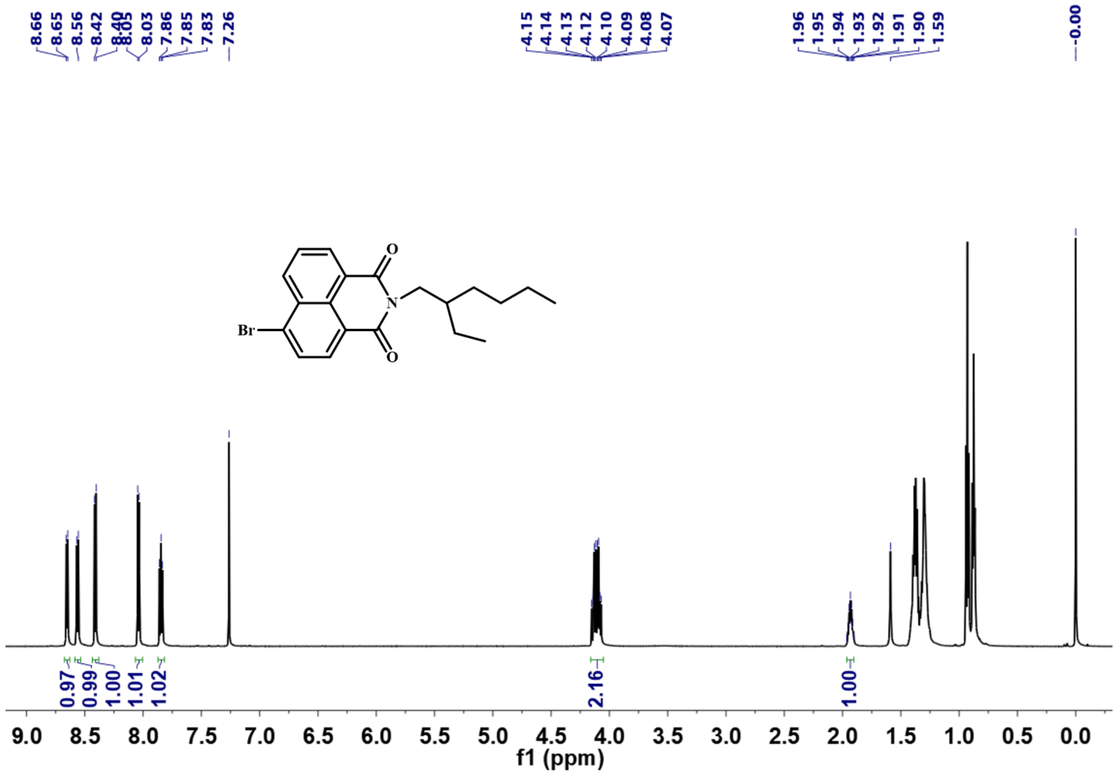

Figure S3. ^{1}H NMR spectra of Br-NAP.

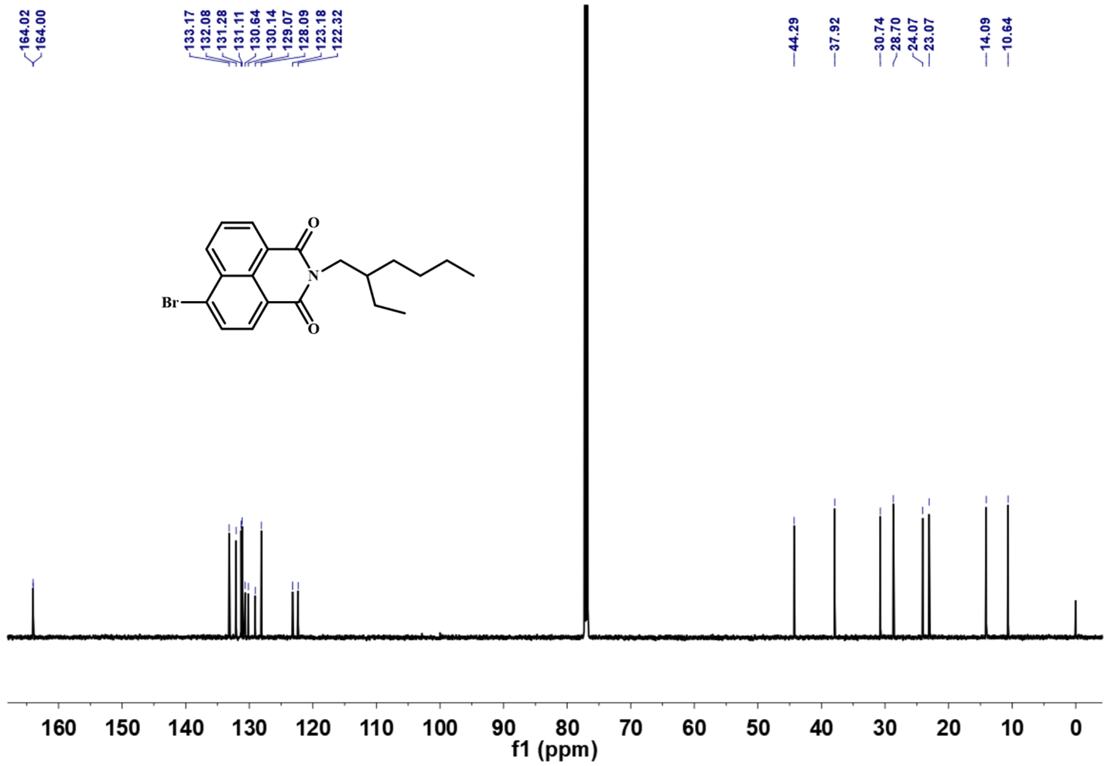

Figure S4. ^{13}C NMR spectra of Br-NAP

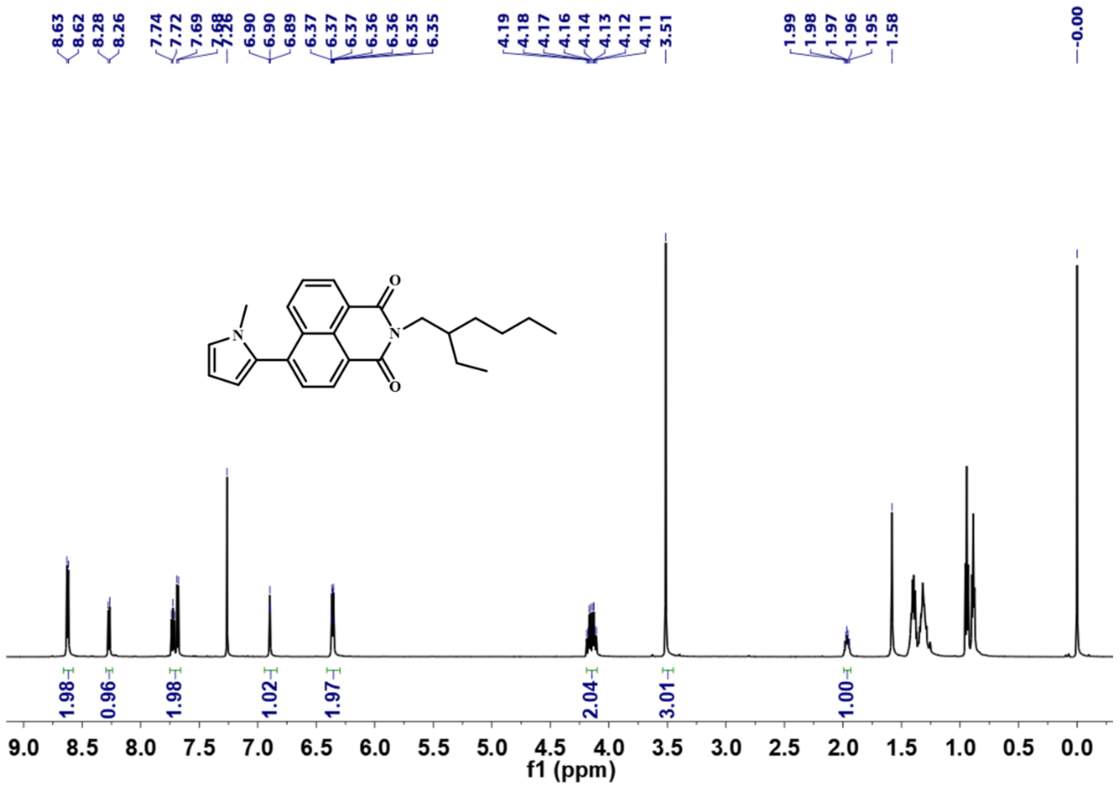

Figure S5. ^{1}H NMR spectra of MP-NAP

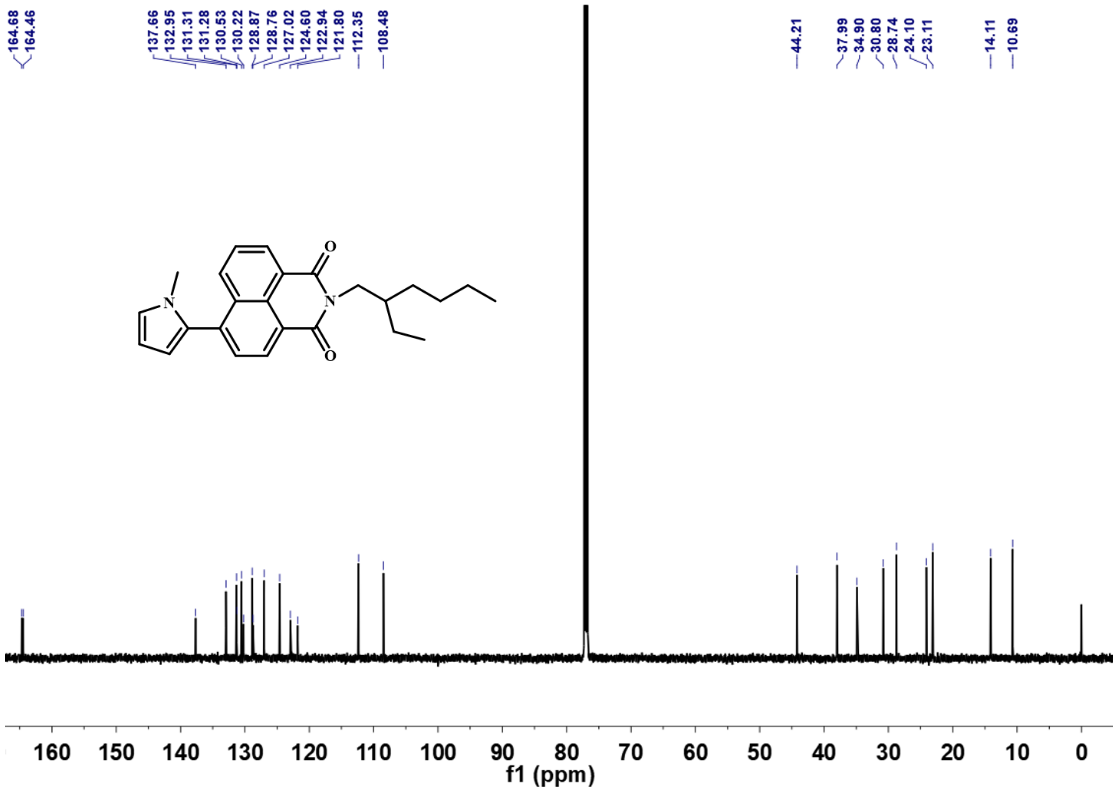

Figure S6. ^{13}C NMR spectra of MP-NAP

2.2 Synthesis of DMA-NAP

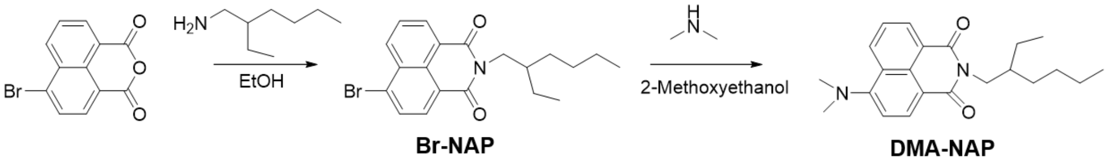

Scheme S2. Synthesis route for DMA-NAP.

DMA-NAP was synthesized referring to the reported work.[2] 235 μL (3.5 mmol, 5 eq.) dimethylamine was added dropwise to a solution of 267.9 mg (0.69 mmol, 5 eq.) Br-NAP in 5 mL 2-methoxyethanol. The mixture was heated to reflux for 2 h, while monitored by TLC. After completion, the reaction was cooled to room temperature and concentrated under reduced pressure. The crude product was purified via column chromatography using petrol ether/ethyl acetate (10:1) on a silica gel column. 90 mg of DMA-NAP was obtained as yellow solids with a yield of 73%. HRMS calculated for $[C_{22}H_{29}N_2O_2]^+$: 353.2224; found: 353.2218.

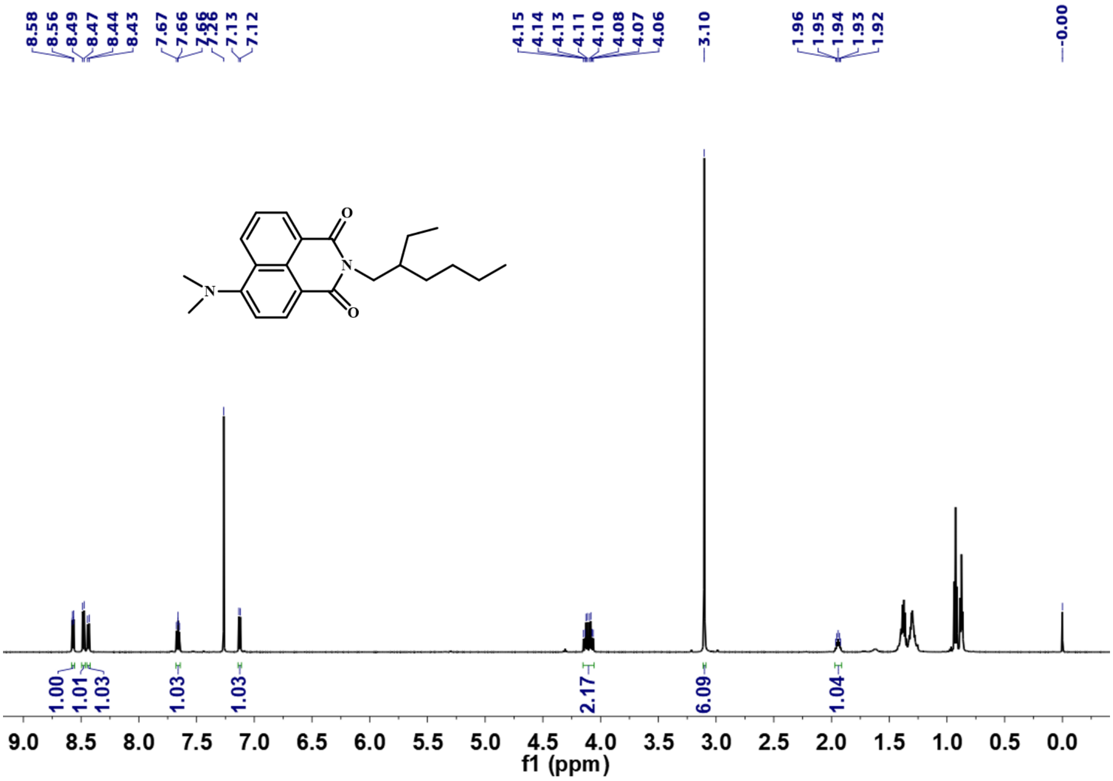

Figure S7. ^1H NMR spectra of DMA-NAP.

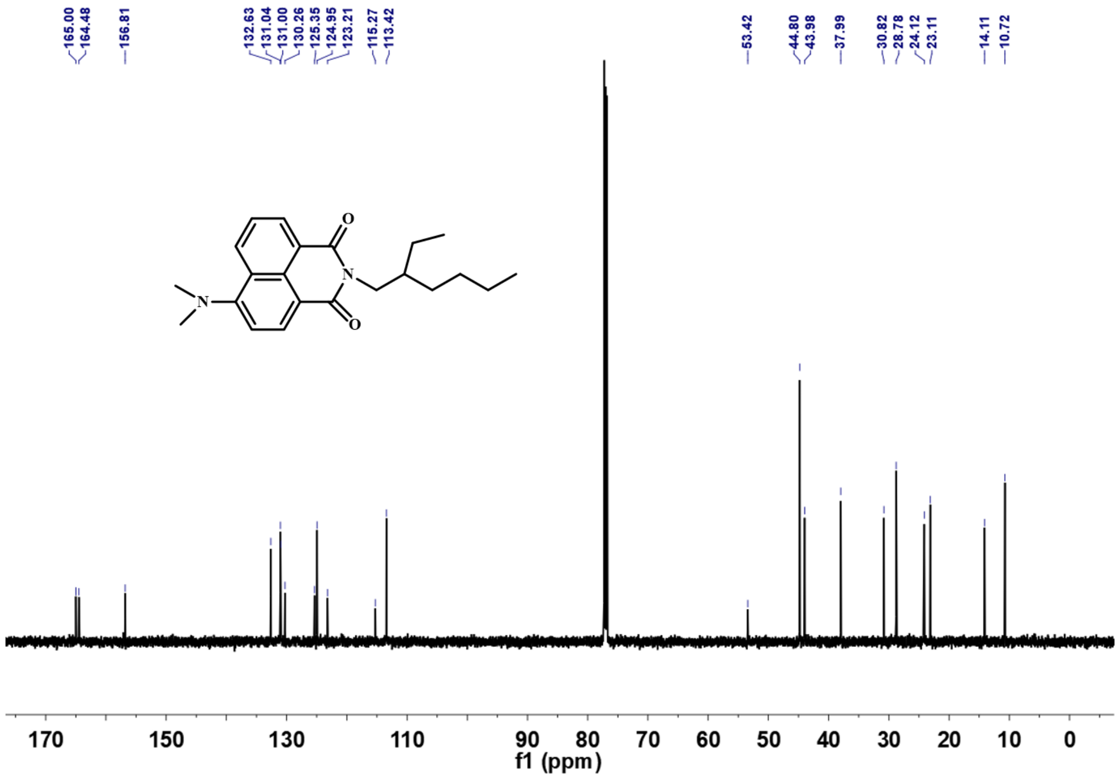

Figure S8. ^{13}C NMR spectra of DMA-NAP.

2.3 Synthesis of HP-NAP

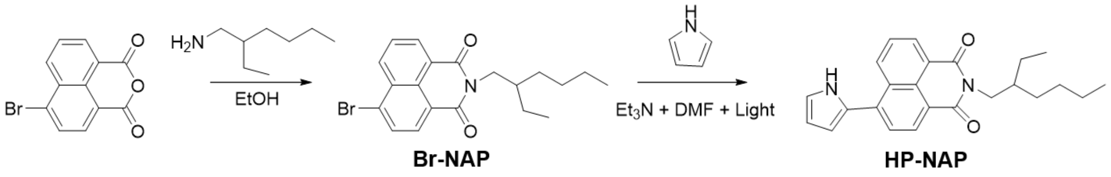

Scheme S3. Synthesis route for HP-NAP.

The synthesis procedure for HP-NAP is similar to that of MP-NAP, except that 1.8 mL pyrrole was used instead of N-methylpyrrole. HP-NAP was obtained as orange-yellow solids with a yield of ~70%. HRMS calculated for $[C_{24}H_{27}N_2O_2]^+$: 375.2067; found: 375.2065.

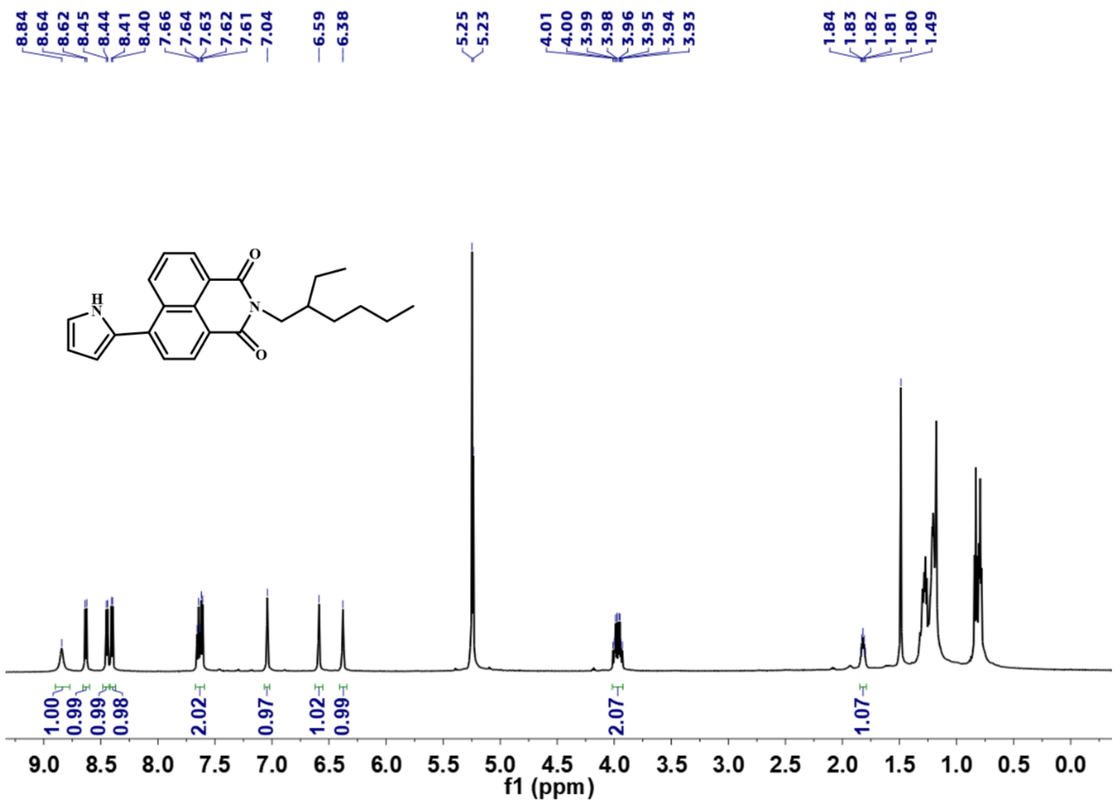

Figure S9. ^{1}H NMR spectra of HP-NAP.

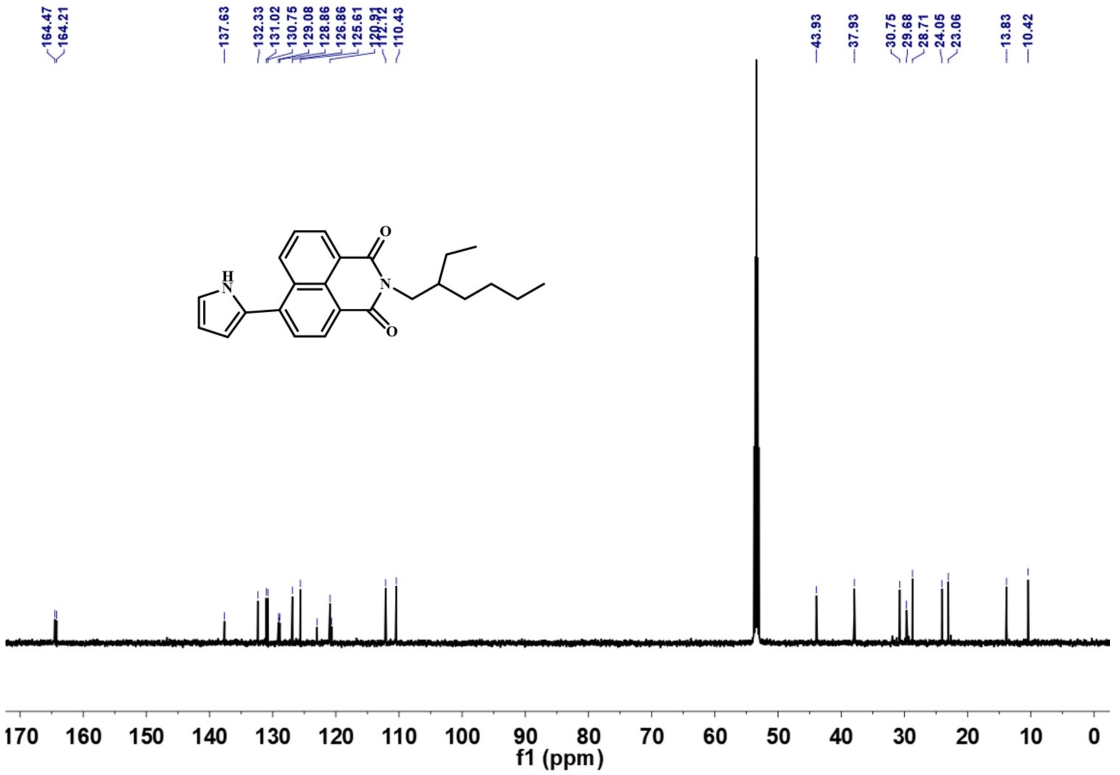

Figure S10. ^{13}C NMR spectra of HP-NAP.

3. Spectroscopic and bioimaging studies of DMA-NAP, HP-NAP, and MP-NAP

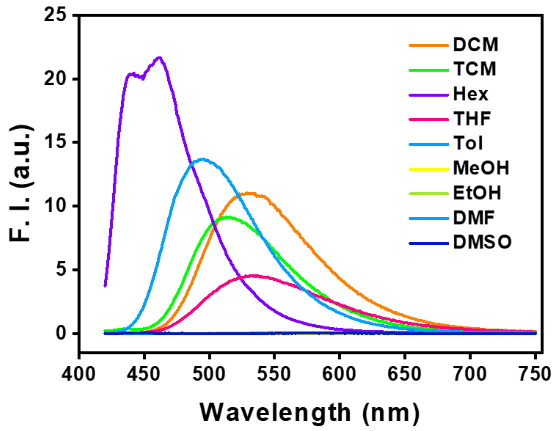

Figure S11. Fluorescence emission spectra of MP-NAP in different solvents. Concentration: 2.5×10^{-5} M. Hex: hexane; Tol: toluene; THF: tetrahydrofuran; DCM: dichloromethane; TCM: trichloromethane; MeOH: methanol; EtOH: ethanol; DMF: dimethylformamide; DMSO: dimethylsulfoxide. λ_{ex} = 400 nm.

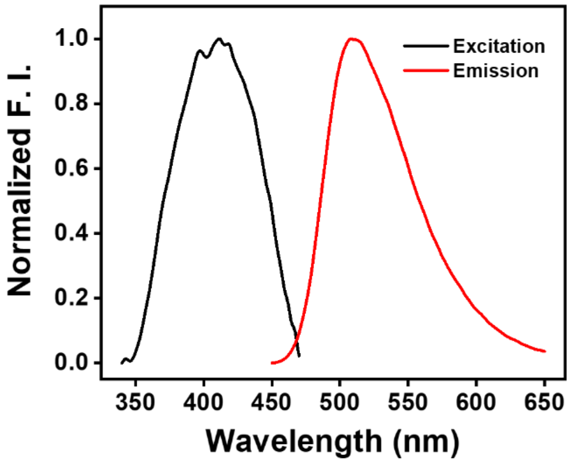

Figure S12. Normalized fluorescence excitation (black) and emission (red) spectra of DMA-NAP in dichloromethane. Concentration = 3.0×10^{-5} M; λ_{ex} = 415 nm; λ_{em} = 510 nm.

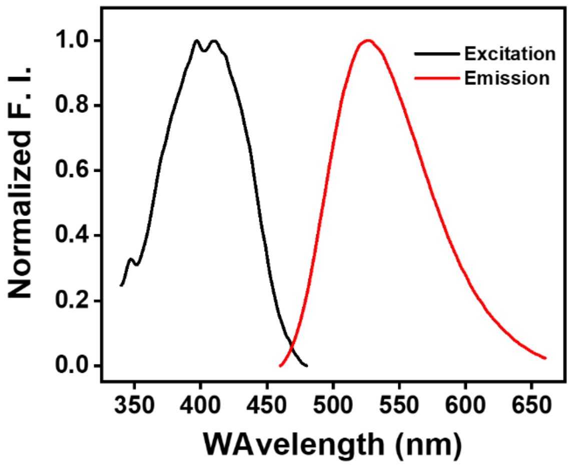

Figure S13. Normalized fluorescence excitation (black) and emission (red) spectra of HP-NAP in dichloromethane. Concentration = 2.5×10^{-5} M; λ_{ex} = 410 nm; λ_{em} = 528 nm.

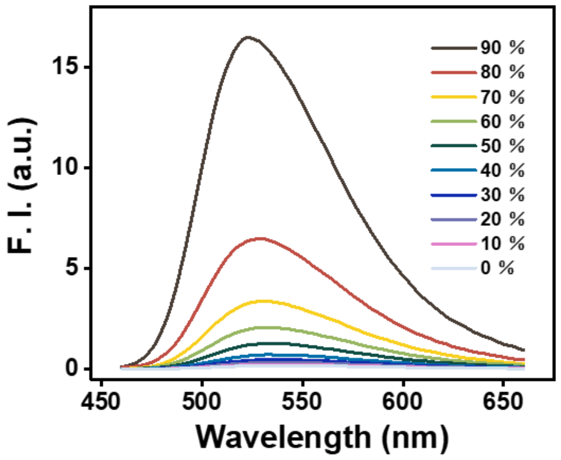

Figure S14. Fluorescence emission spectra of DMA-NAP in the mixtures of DCM and MeOH with varied volume fractions of DCM. DCM: dichloromethane; MeOH: methanol. Concentration = 3.0×10^{-5} M; λ_{ex} = 415 nm.

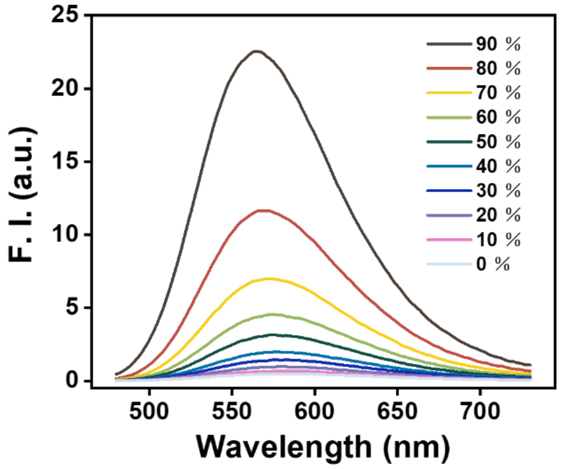

Figure S15. Fluorescence emission spectra of HP-NAP in the mixtures of DCM and MeOH with varied volume fractions of DCM. DCM: dichloromethane; MeOH: methanol. Concentration = 2.5×10^{-5} M; λ_{ex} = 410 nm.

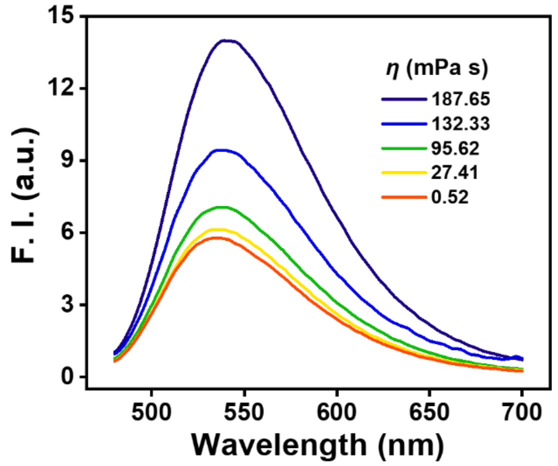

Figure S16. Fluorescence emission spectra of DMA-NAP in the mixtures of methanol and glycerol with different viscosities. Concentration = 3.0×10^{-5} M; λ_{ex} = 415 nm.

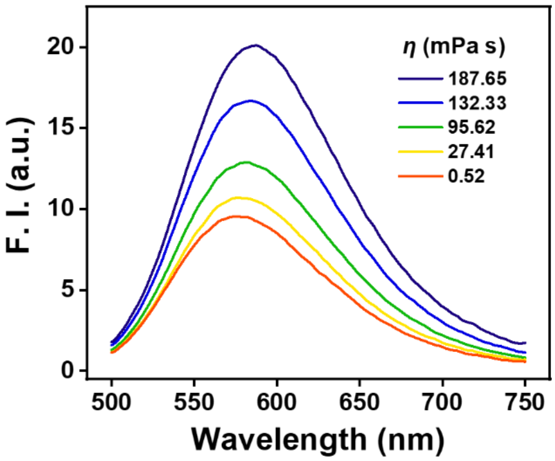

Figure S17. Fluorescence emission spectra of HP-NAP in the mixtures of methanol and glycerol with different viscosities. Concentration = 2.5×10^{-5} M; λ_{ex} = 410 nm.

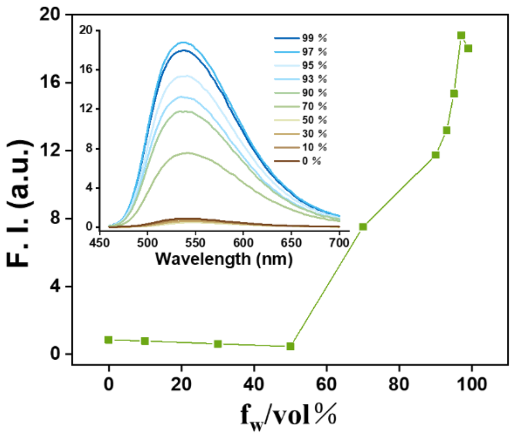

Figure S18. Fluorescence emission spectra of DMA-NAP in the mixtures of H_2O and MeOH with varied volume fractions of water. Concentration = 3.0×10^{-5} M; λ_{ex} = 415 nm.

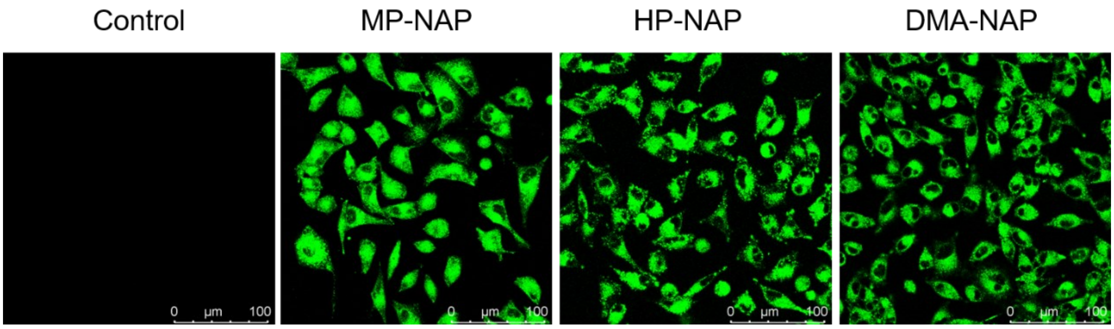

Figure S19. Fluorescence imaging of control cells (without dyes) and live cells treated with MP-NAP, HP-NAP, and DMA-NAP. Dye concentration: 10 μg/mL; incubation time: 4 hours at 37 °C; 5% CO_2.

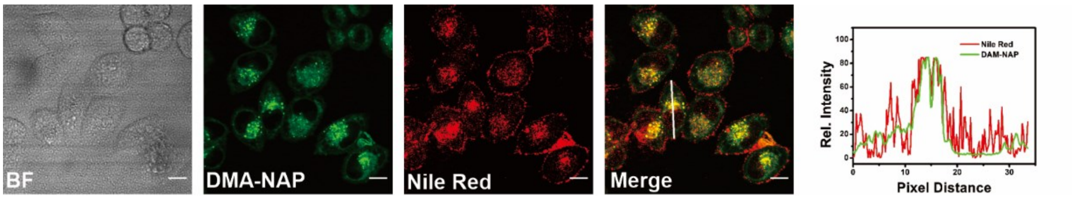

Figure S20. Fluorescence imaging Confocal microscopy of live HeLa cells co-stained with DMA-NAP and Nile Red; the right panel shows the intensity profile along the white line highlighted in the left panel. [DMA-NAP] = 10 μM; [Nile Red] = 1 μM. Excitation wavelength = 488 nm, emission filter = 490-590 nm for DMA-NAP; excitation wavelength = 543 nm, emission filter = 575-620 nm for Nile Red. Scale bars = 20 μm.

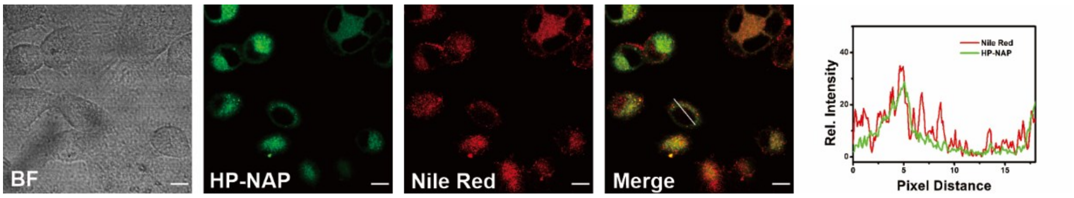

Figure S21. Fluorescence Confocal microscopy of live HeLa cells co-stained with HP-NAP and Nile Red; the right panel shows the intensity profile along the white line highlighted in the left panel. [HP-NAP] = 10 μM; [Nile Red] = 1 μM. Excitation wavelength = 488 nm, emission filter = 490-590 nm for HP-NAP; excitation wavelength = 543 nm, emission filter = 575-620 nm for Nile Red. Scale bars = 20 μm.

Table S1. Pearson's coefficients during the colocalization experiments of lipid droplets using DMA-NAP/HP-NAP/MP-NAP and Nile Red.[a]

Compound	Pearson's coefficient
DMA-NAP	0.33
HP-NAP	0.45
MP-NAP	0.75

[a] During the experiments of imaging lipid droplets, we noted that Nile Red generated significant background fluorescence outside of lipid droplets (Figure 21). The same observations were also reported by Xu *et al.*[3] Similarly, we noted that DMA-NAP and HP-NAP also afforded considerable background fluorescence (Figures S20 and S21). In contrast, MP-NAP led to sharp fluorescence imaging of lipid droplets (Figure 21). The overlapping between the fluorescence signals of naphthalimide derivatives and Nile Red was quantified by the Pearson's coefficient. The Person's coefficients are low to moderate. Notably, this coefficient of MP-NAP (0.75) is significantly higher than those of DMA-NAP (0.33) and HP-NAP (0.45), reflecting the higher selectivity to lipid droplets of MP-NAP, than that of DMA-NAP or HP-NAP.

4. Calculations of DMA-PHA, MP-PHA, DMA-COU, MP-COU, DEA-RHO, and MP-RHO

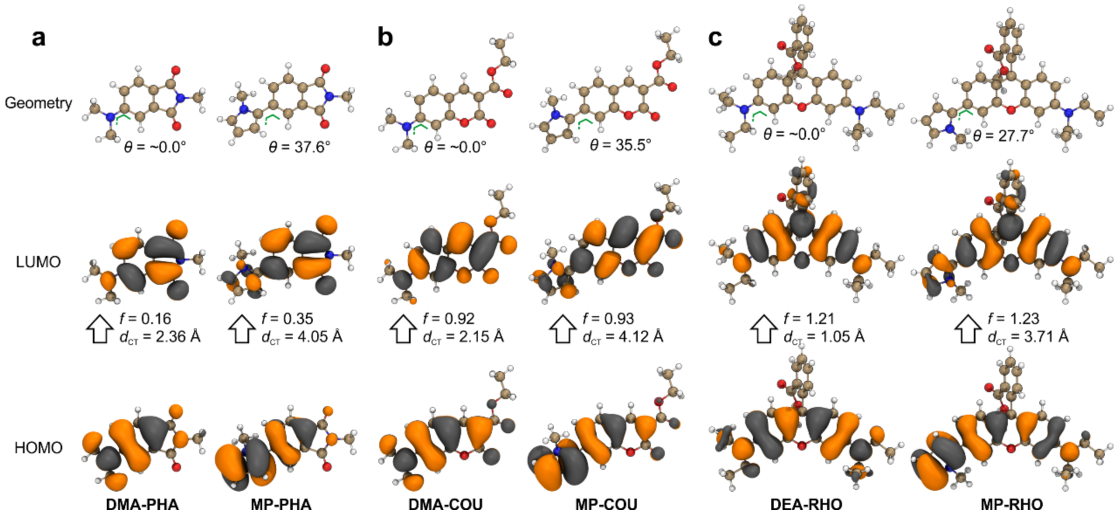

Figure S22. Molecular geometry and the distribution of HOMO and LUMO of (a) DMA-PHA and MP-PHA, (b) DMA-COU and MP-COU, and (c) DEA-RHO and MP-RHO at the FC state calculated at the M062X/def2-SVP level in toluene. The oscillator strength and CT distance are labelled in the inset.

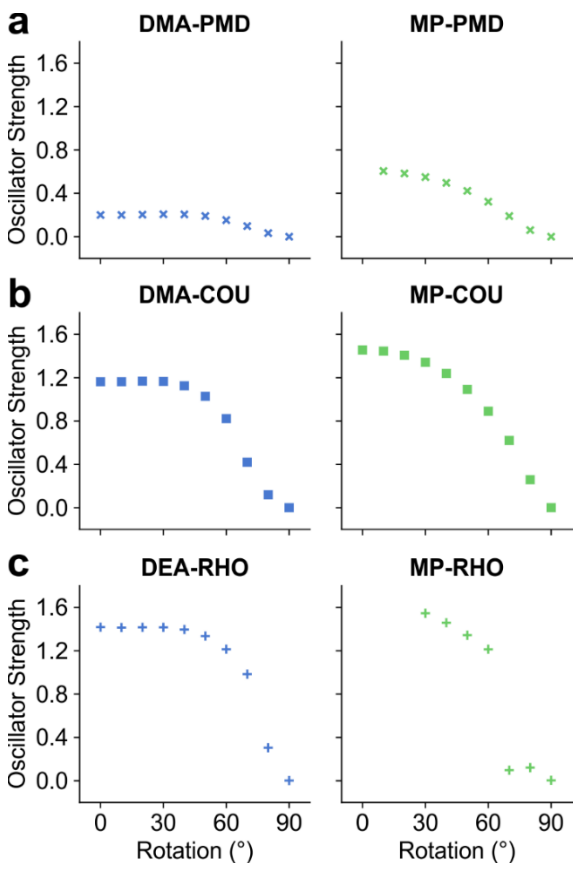

Figure S23. Oscillator strength of (a) DMA-PHA and MP-PHA, (b) DMA-COU and MP-COU, and (c) DEA-RHO and MP-RHO as a function of rotation angle (θ) on the S_1 PES for TICT calculated at the M062X/def2-SVP/cLR-SMD level in DMSO.

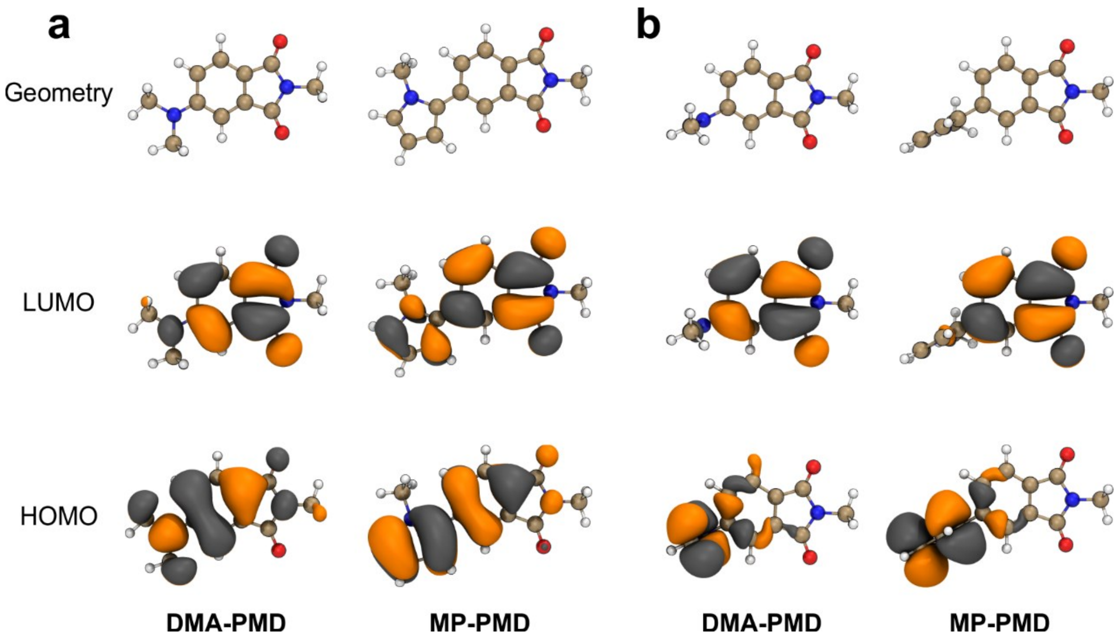

Figure S24. Molecular geometry and the distribution of HOMO and LUMO of DMA-PMD and MP-PMD at the (a) LE/ICT and (b) TICT states calculated at the M062X/def2-SVP/cLR-SMD level in DMSO.

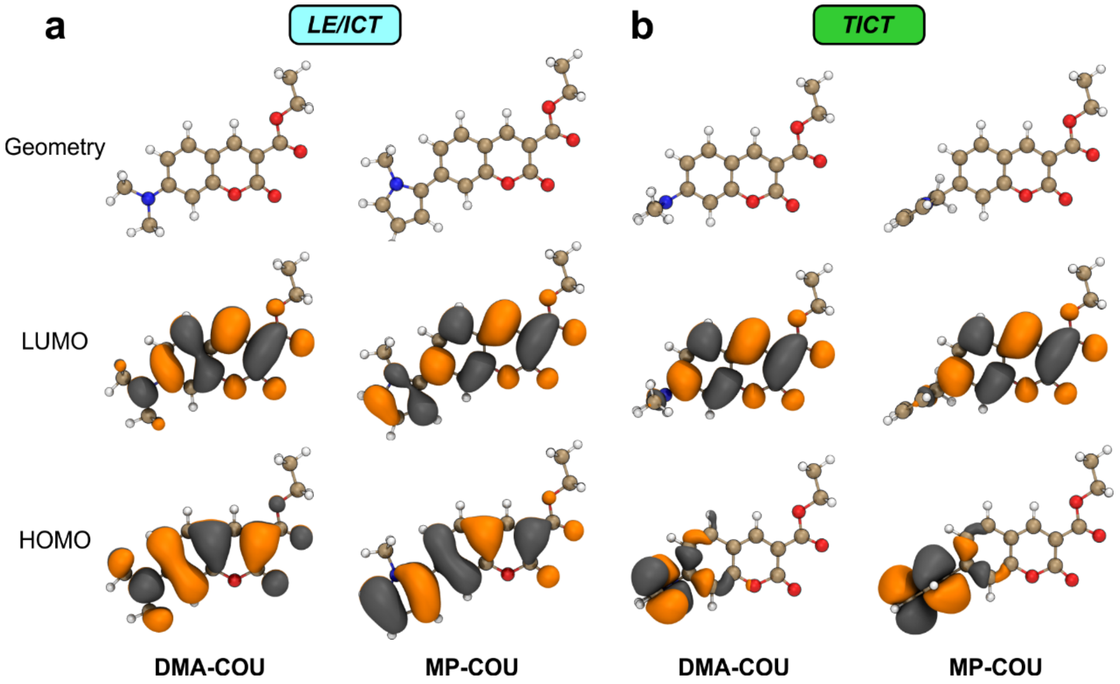

Figure S25. Molecular geometry and the distribution of HOMO and LUMO of DMA-COU and MP-COU at the (a) LE/ICT and (b) TICT states calculated at the M062X/def2-SVP/cLR-SMD level in DMSO.

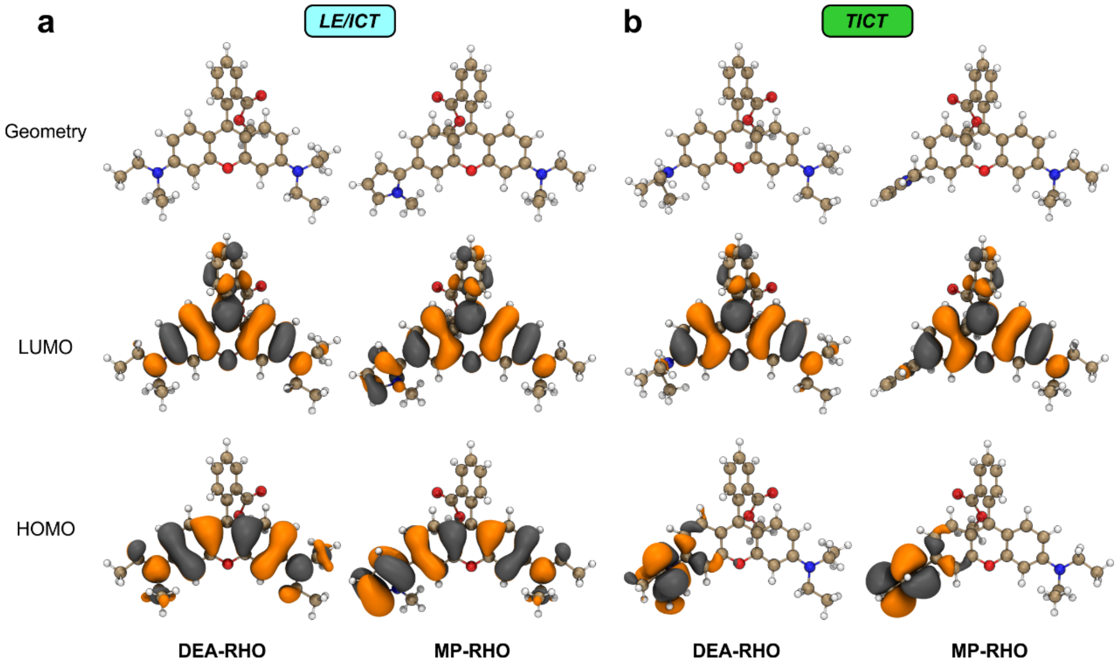

Figure S26. Molecular geometry and the distribution of HOMO and LUMO of DEA-RHO and MP-COU at the (a) LE/ICT and (b) TICT states calculated at the M062X/def2-SVP/cLR-SMD level in DMSO.

5. Synthesis of MP-PHA, MP-COU, and MP-RHO

5.1 Synthesis of MP-PHA

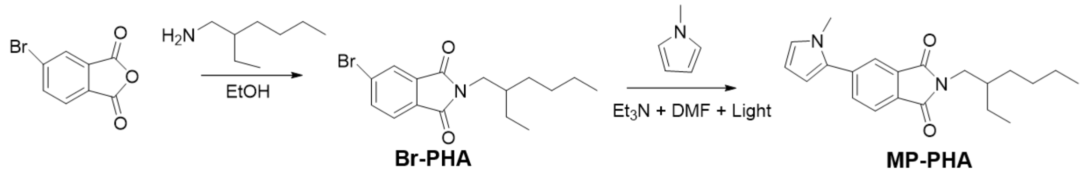

Scheme S4. Synthesis route for MP-PHA.

1.0 g of 4-bromo-phthalimide (4.4 mmol) was dissolved in 35 mL glacial acetic acid and 0.5 mL of 2-ethylhexylamine was added into the above solution under stirring at room temperature. Then the mixture was heated to reflux. After 2 hours, the reaction was stopped and the precipitate was collected by filtration. Br-PHA (850 mg, white solid) was obtained by repeated washing (water) of the precipitate. The synthesis procedure for MP-PHA was similar to MP-NAP, except that 0.6 mmol (202 mg) of Br-PHA was used instead of Br-NAP. MP-PHA was obtained as green solids with a yield of 75%. HRMS calculated for $[C_{13}H_{13}NO_2Na]^+$: 361.1892; found: 361.1894.

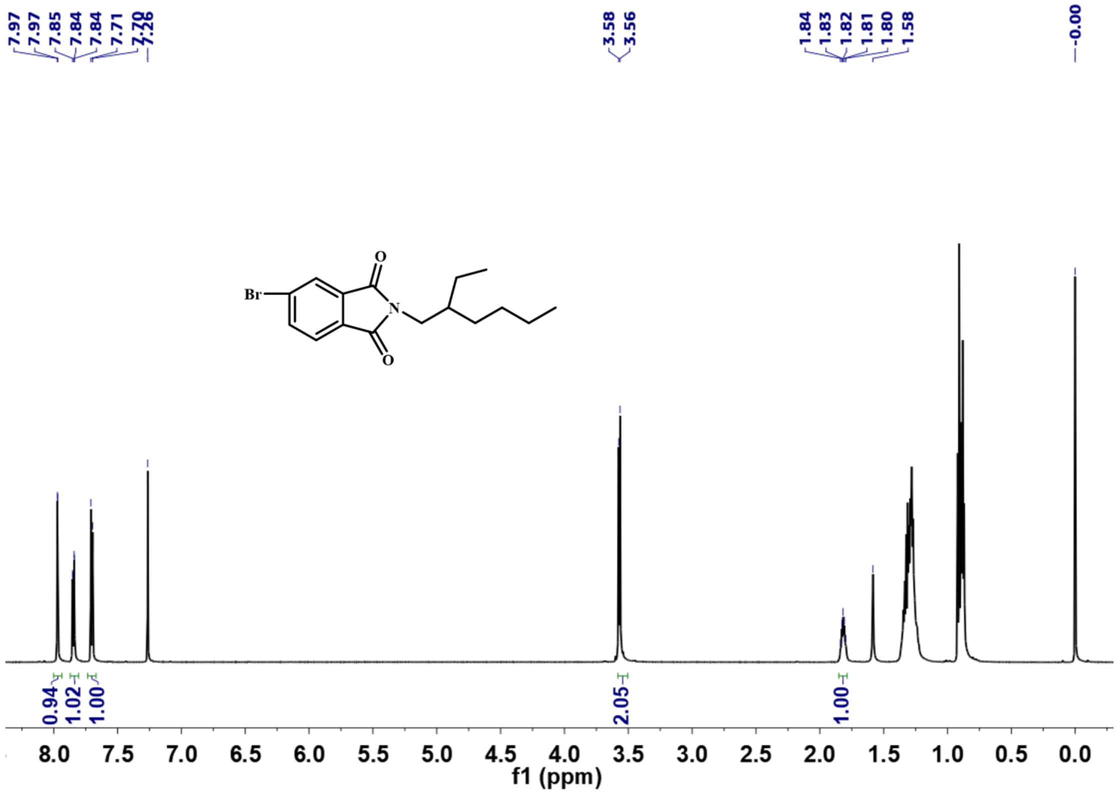

Figure S27. ^{1}H NMR spectra of Br-PHA.

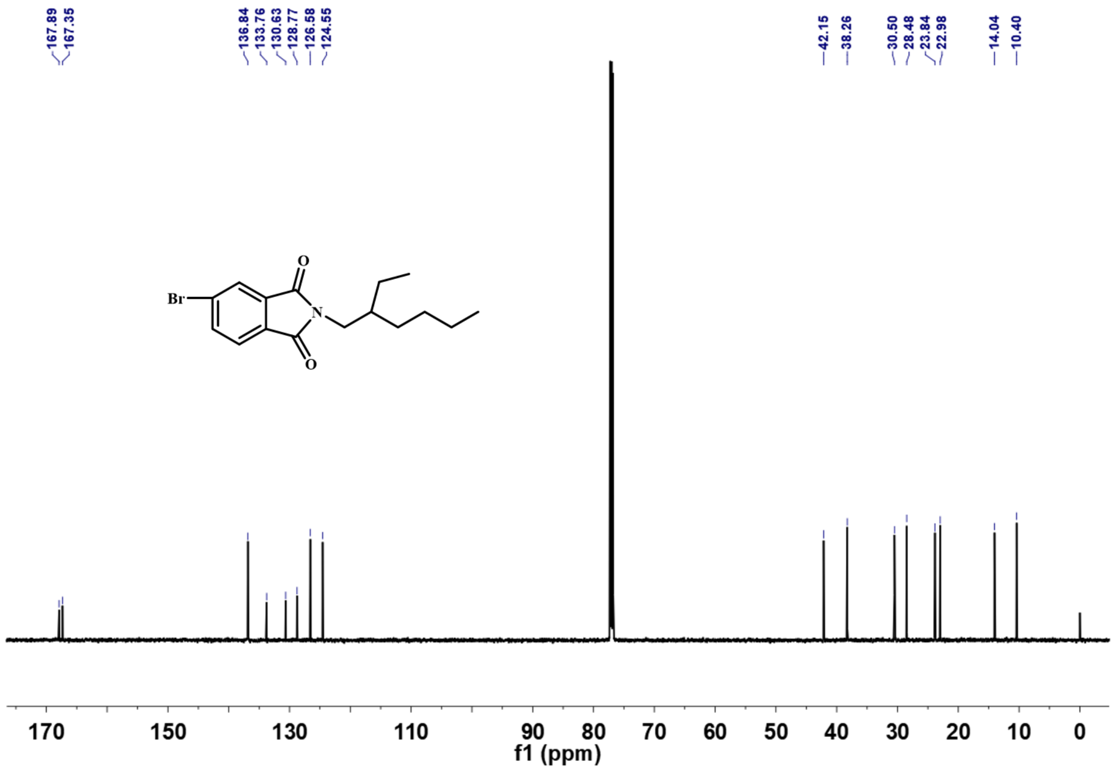

Figure S28. ^{13}C NMR spectra of Br-PHA.

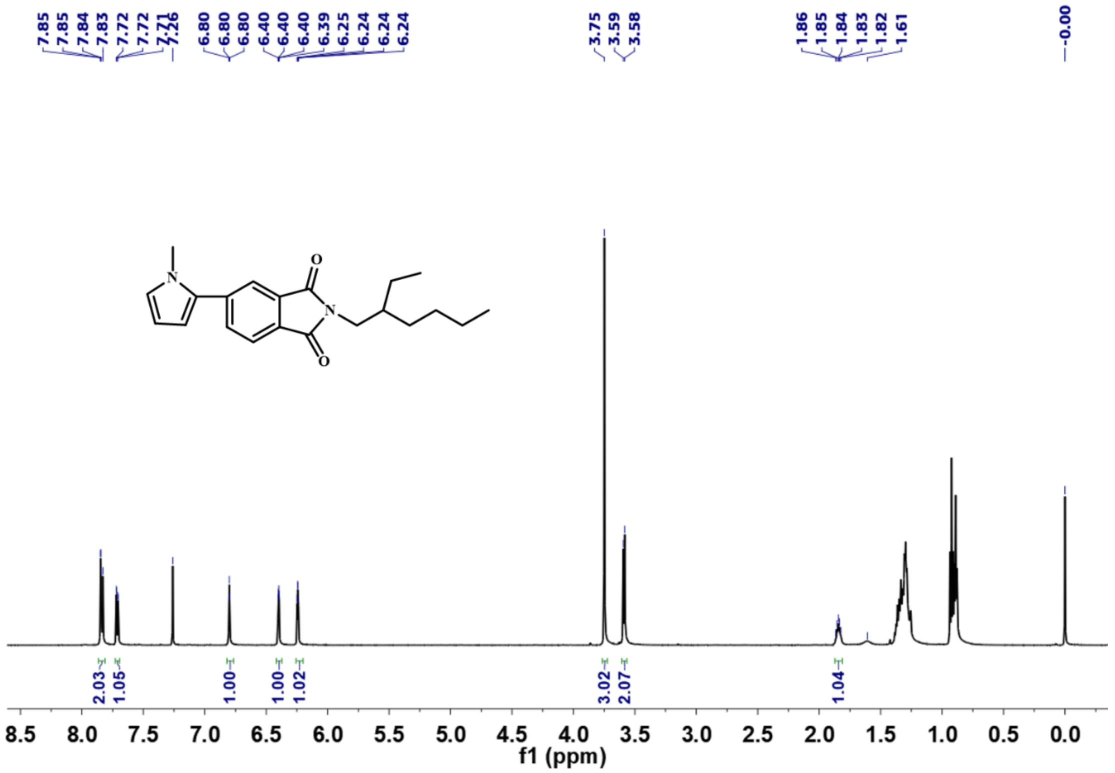

Figure S29. ^{1}H NMR spectra of MP-PHA.

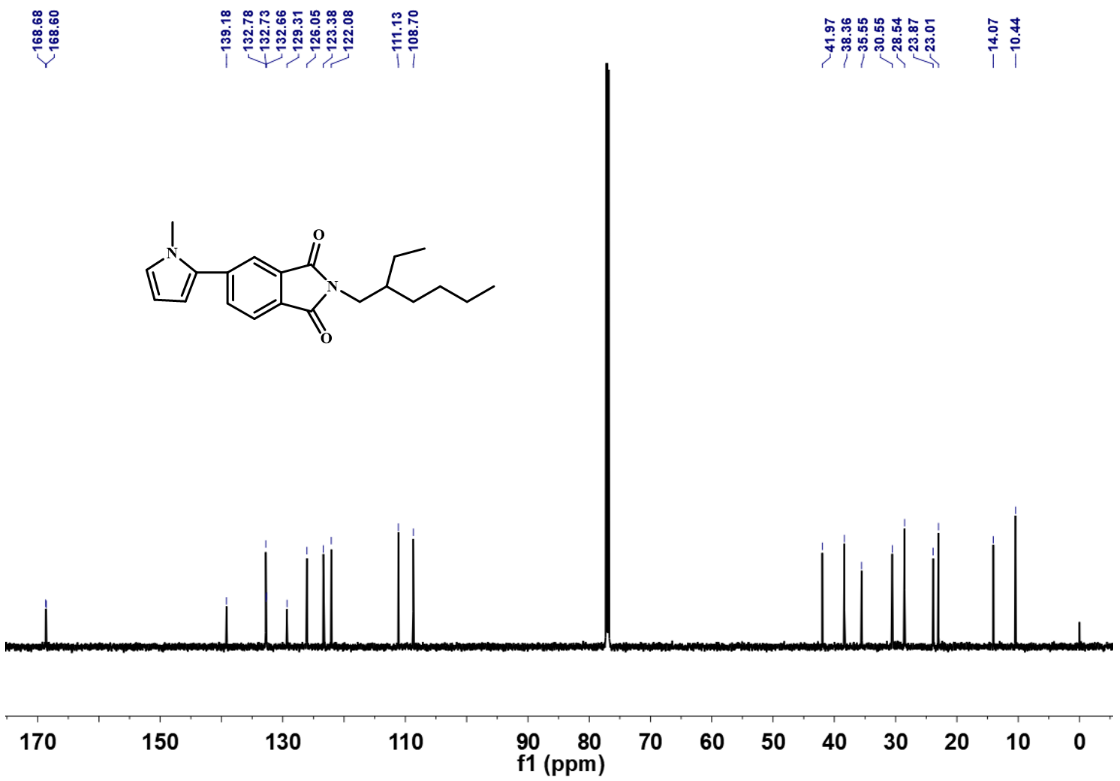

Figure S30. ^{13}C NMR spectra of MP-PHA.

5.2 Synthesis of MP-COU

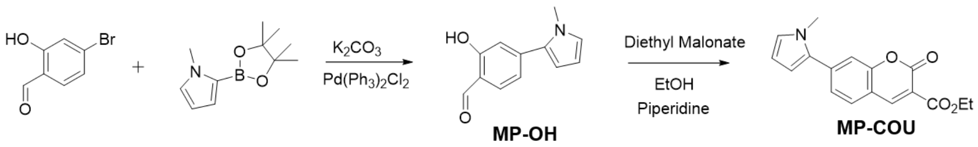

Scheme S5. Synthesis route for MP-COU.

A 100 mL two-neck flask was charged with 4-Bromo-2-hydroxybenzaldehyde (0.3, 1.5 mmol), potassium carbonate (0.5 g, 3.6 mmol), and N-Methilpyrrole-2-Boronic Acid Pinacol ester (0.416 g, 1.6 mmol) under nitrogen. Afterwards, THF (20 mL), H_2O (20 mL) and $PdCl_2(PPh_3)_2$ (105 mg, 0.015 mmol) were added to the mixture, and the reaction mixture was allowed to stir at 75 °C for 12 hours under nitrogen. When the reaction was completed, DCM (5 mL) and H_2O (5 mL) were added to the mixture. The aqueous layer was extracted with DCM (3×20 mL). The organic layer was collected and dried with anhydrous Na_2SO_4 overnight. Then the solvent was removed by rotary evaporation. The crude product was purified via column chromatography using petrol ether/ethyl acetate (10:1) on a silica gel column. 130 mg (0.64 mmol) of MP-OH solids

were obtained (yellowish-brown) and it was suspended in EtOH (10 mL).

Diethyl malonate (195 μL, 1.28 mmol) and piperidine (18 μL, 0.19 mmol) were added, and the reaction was stirred at reflux for 12 h. It was then cooled to room temperature and concentrated under reduced pressure. The crude product was purified via column chromatography using petrol ether/ethyl acetate (5:1) on a silica gel column. 123 mg of MP-COU was obtained as yellow solids with a yield of 65%. HRMS calculated for $[C_{17}H_{16}NO_4]^+$: 298.1035; found: 298.1087.

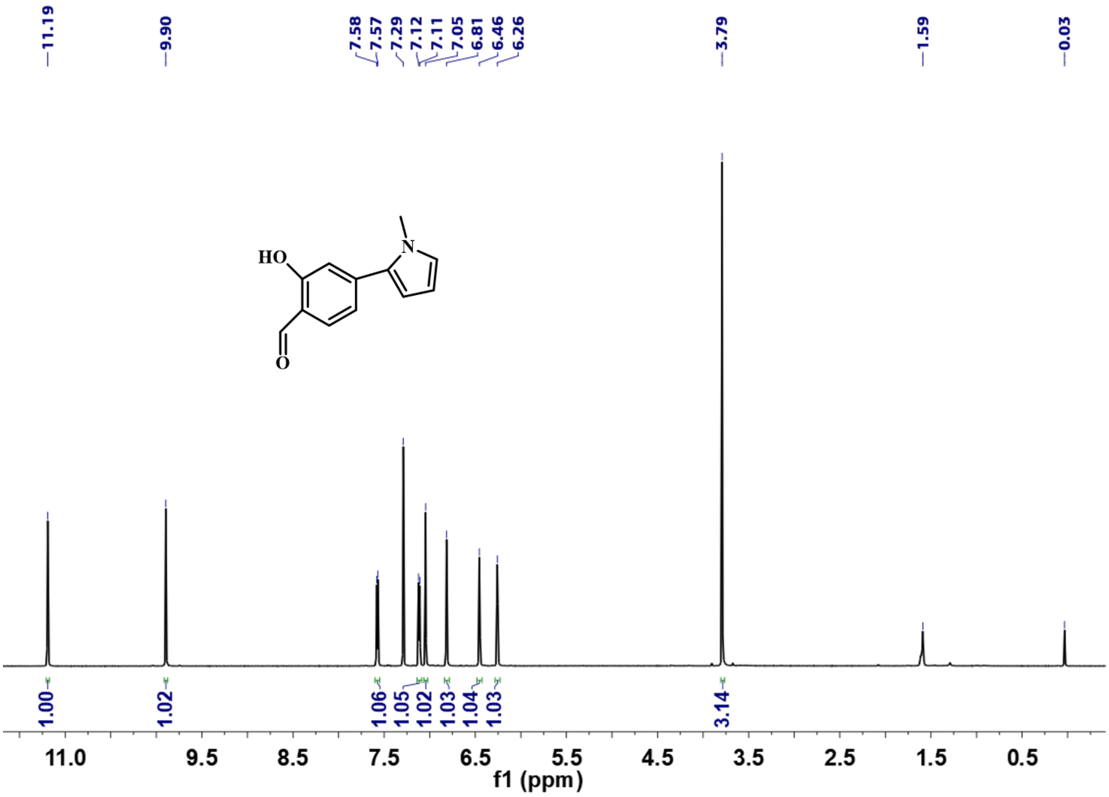

Figure S31. ^{1}H NMR spectra of MP-OH.

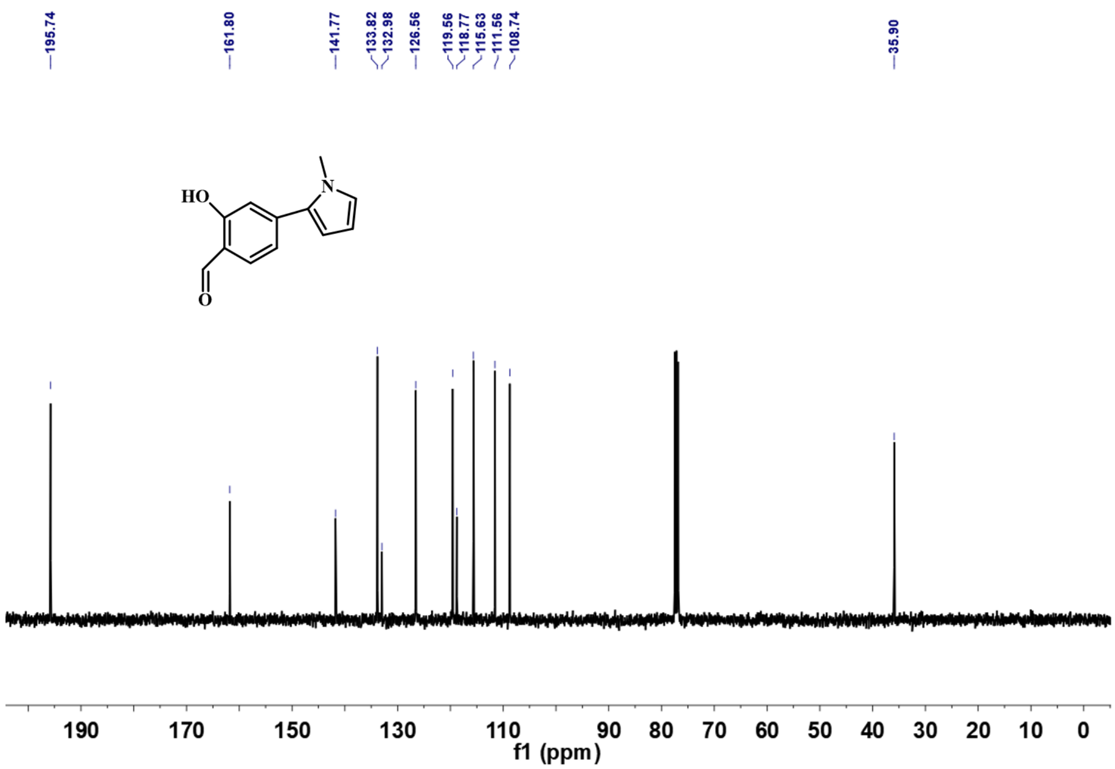

Figure S32. ^{13}C NMR spectra of MP-OH.

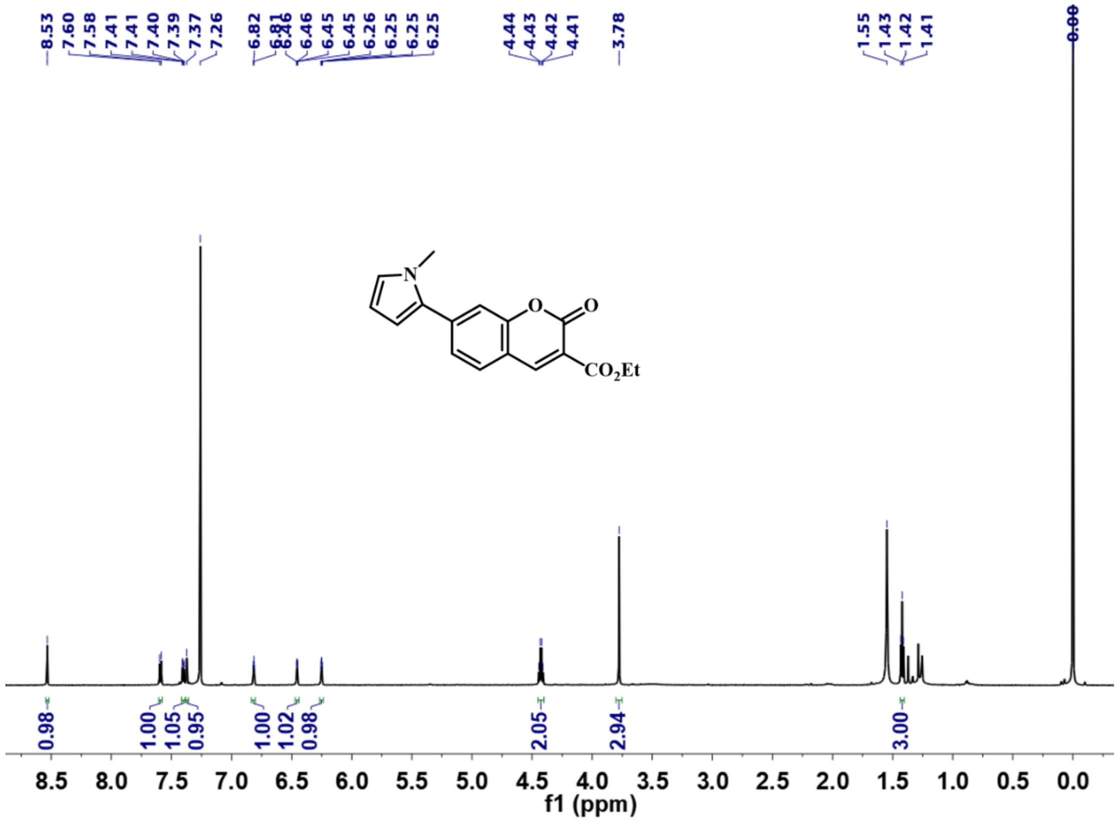

Figure S33. ^{1}H NMR spectra of MP-COU.

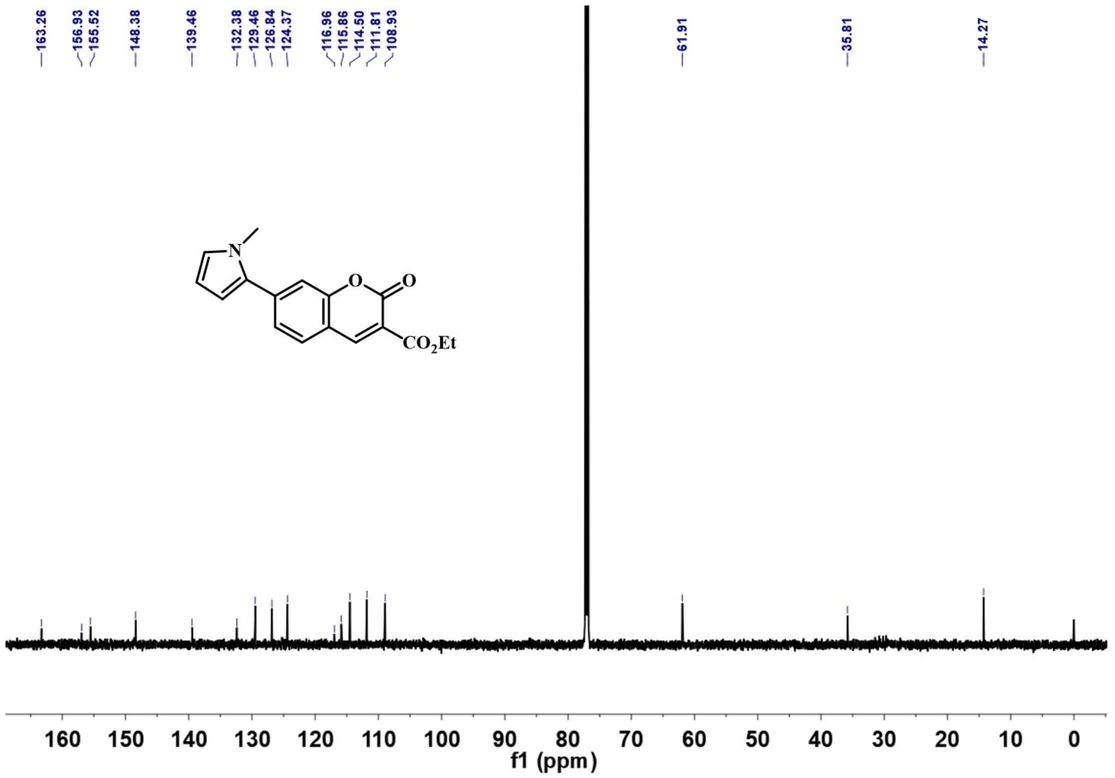

Figure S34. ^{13}C NMR spectra of MP-COU.

5.3 Synthesis of MP-RHO

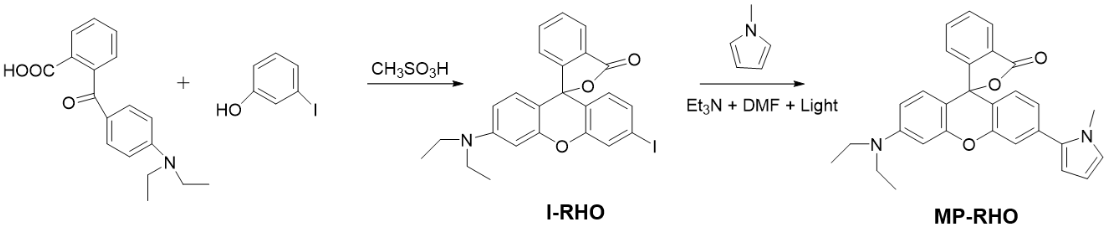

Scheme S6. Synthesis route for MP-RHO.

I-RHO was synthesized referring to the reported work.[6] In detail, 2-(4-diethylamino-2-hydroxybenzoyl) benzoic acid (313 mg, 1.00 mmol) and 3-iodophenol (260 mg, 1.18 mmol) in 10 mL CH_3SO_3H was slowly heated to 150 °C. After 8 hours, saturated Na_2CO_3 was drop added until the pH value of the solution rose to 9. The mixture was then extracted with DCM (3×50 mL). The organic layer was dried with anhydrous Na_2SO_4 overnight and then the solvent was removed by rotary evaporation. The mixture was further purified by column chromatography (SiO_2, DCM) to give light pink solid 380 mg (yield 77%).

The procedure for synthesis of MP-RHO is similar to MP-NAP, except that 0.6 mmol (298 mg) of I-RHO was used instead of Br-NAP. 160 mg MP-RHO was obtained as light purple solids with a yield of 35%. HRMS calculated for $[C_{29}H_{27}N_2O_3]^+$: 451.1977; found: 451.2017.

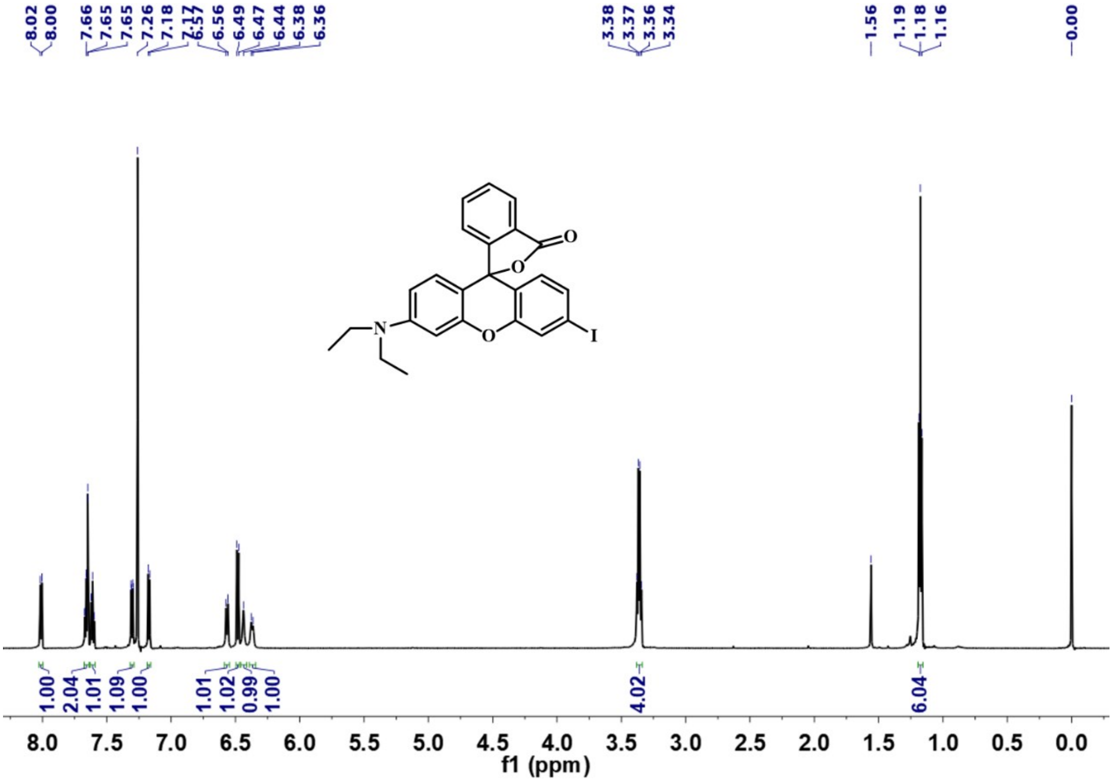

Figure S35. ^{1}H NMR spectra of I-RHO.

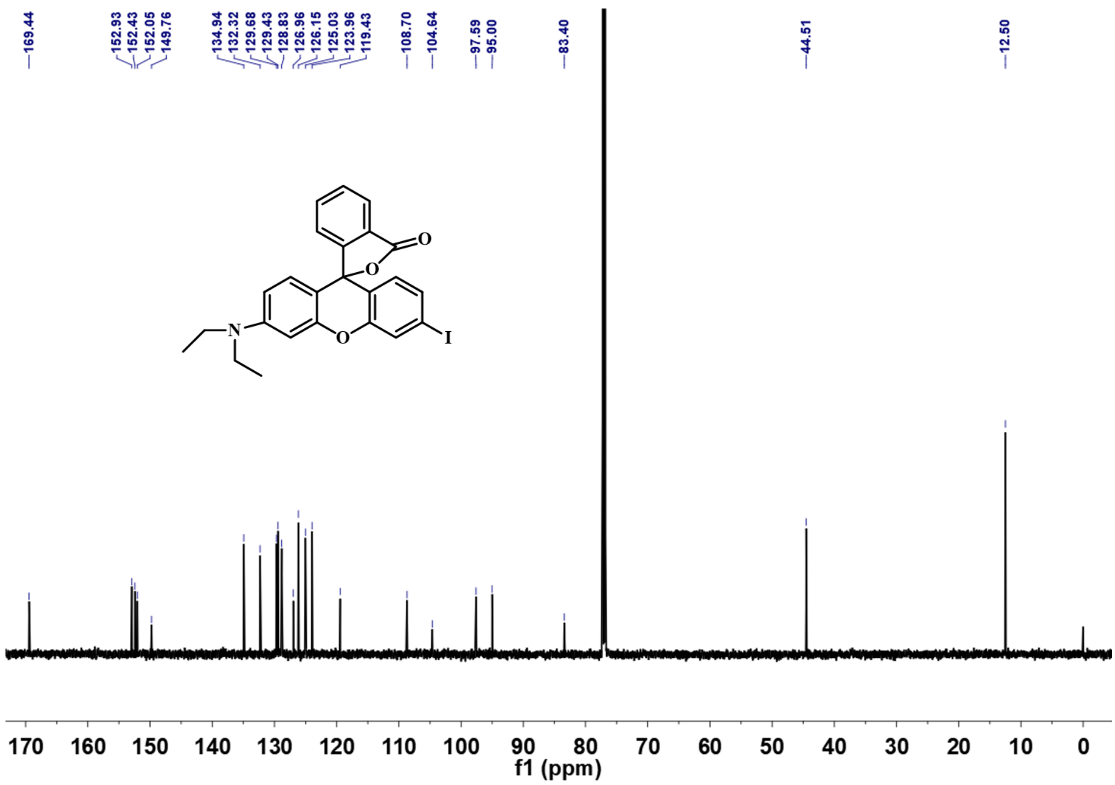

Figure S36. ^{13}C NMR spectra of I-RHO.

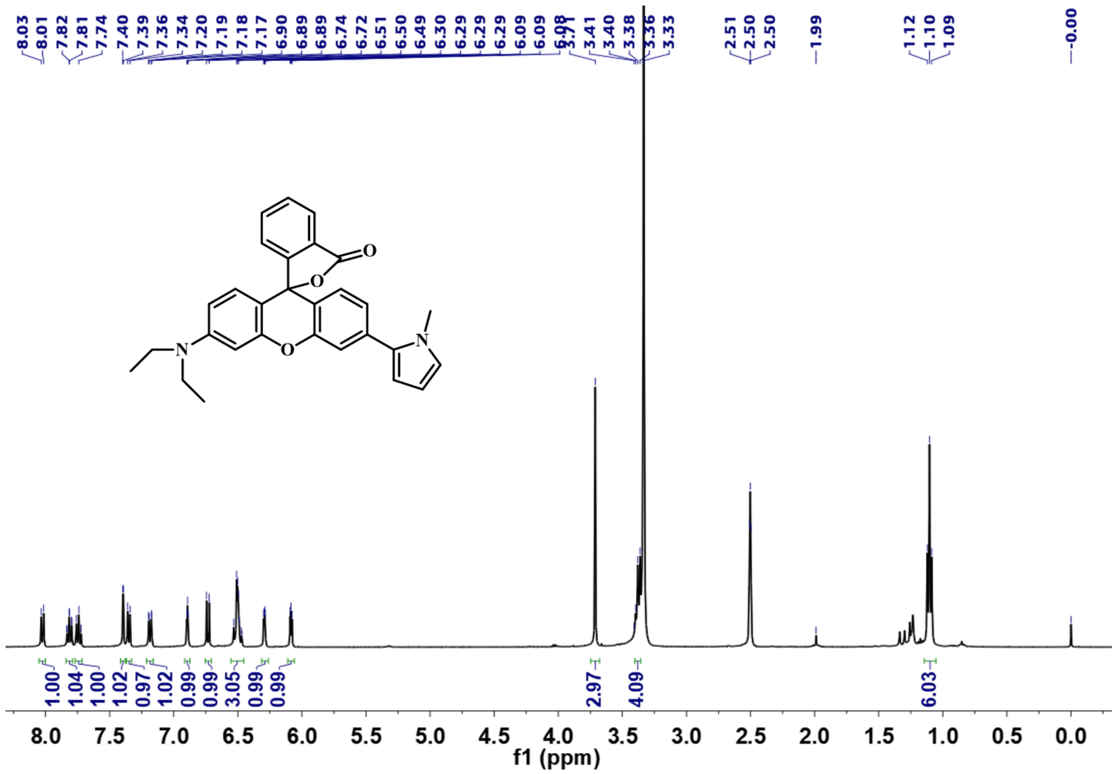

Figure S37. ^{1}H NMR spectra of MP-RHO.

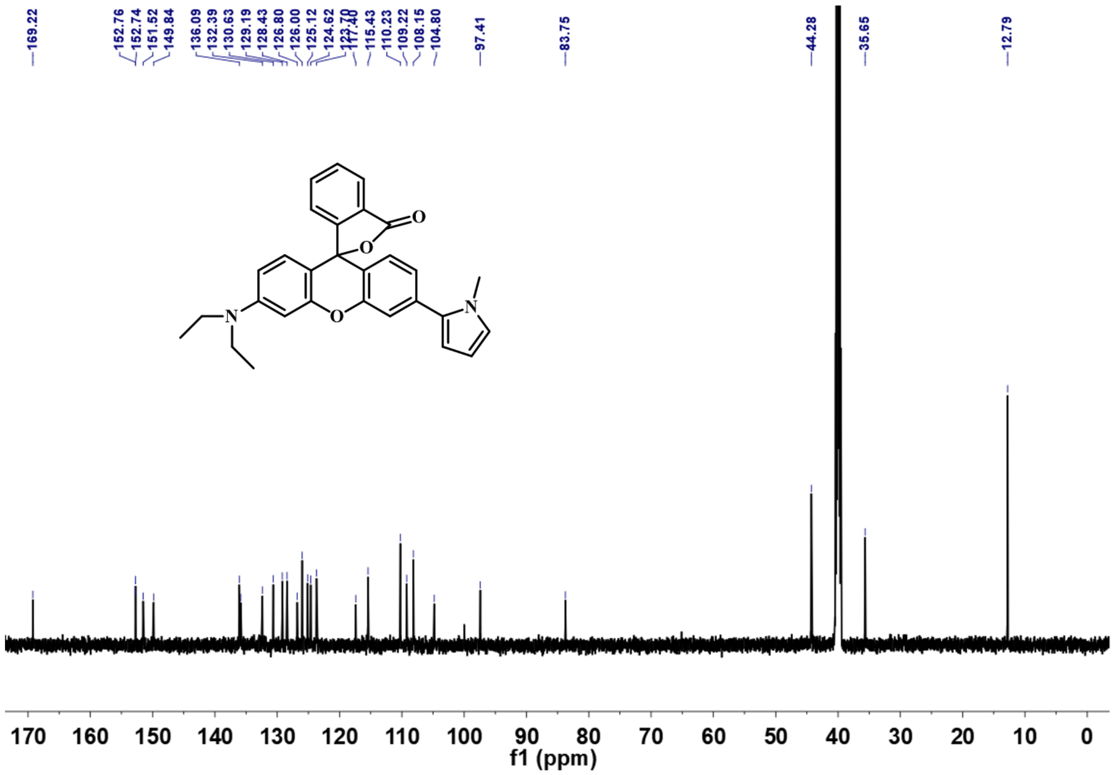

Figure S38. ^{13}C NMR spectra of MP-RHO.

6. Spectroscopic study of DMA-NAP, HP-NAP, and MP-NAP

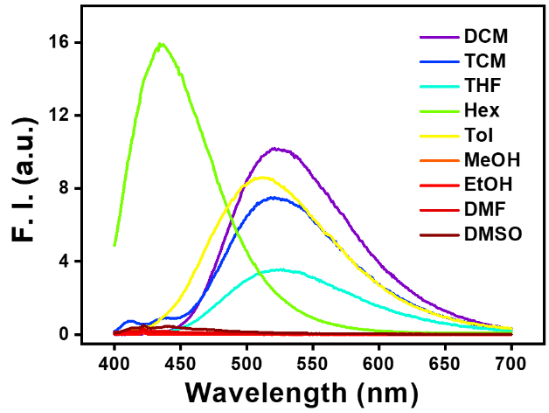

Figure S39. Fluorescence emission spectra of MP-PHA in different solvents. Hex: hexane; Tol: toluene; THF: tetrahydrofuran; DCM: dichloromethane; TCM: trichloromethane; MeOH: methanol; EtOH: ethanol; DMF: dimethylformamide; DMSO: dimethylsulfoxide. Concentration = 2.5×10^{-5} M. λ_{ex} = 375 nm.

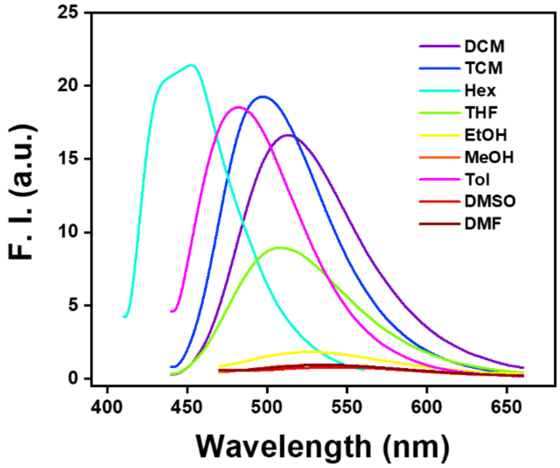

Figure S40. Fluorescence emission spectra of MP-COU in different solvents. Hex: hexane; Tol: toluene; THF: tetrahydrofuran; DCM: dichloromethane; TCM: trichloromethane; MeOH: methanol; EtOH: ethanol; DMF: dimethylformamide; DMSO: dimethylsulfoxide. Concentration = 2.0×10^{-5} M; λ_{ex} = 400 nm.

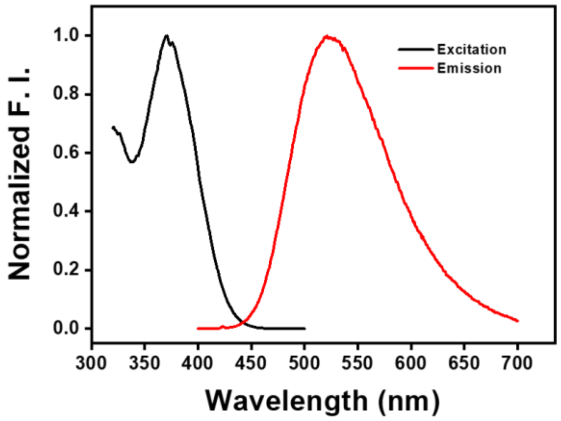

Figure S41. Normalized fluorescence excitation (black) and emission (red) spectra of MP-PHA in dichloromethane. Concentration = 2.5×10^{-5} M; λ_{ex} = 375 nm; λ_{em} = 525 nm.

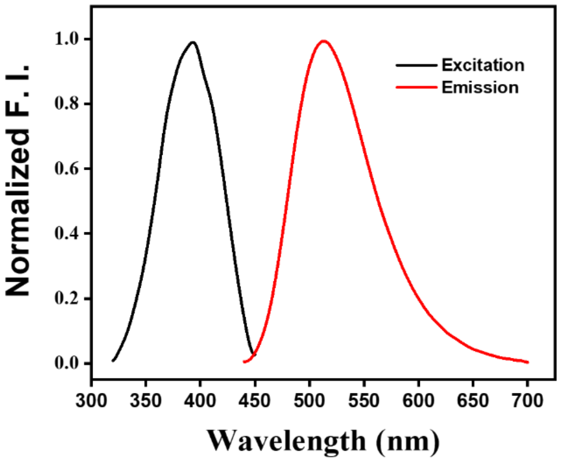

Figure S42. Normalized fluorescence excitation (black) and emission (red) spectra of MP-COU in dichloromethane. Concentration = 2.0×10^{-5} M; λ_{ex} = 400 nm; λ_{em} = 510 nm.

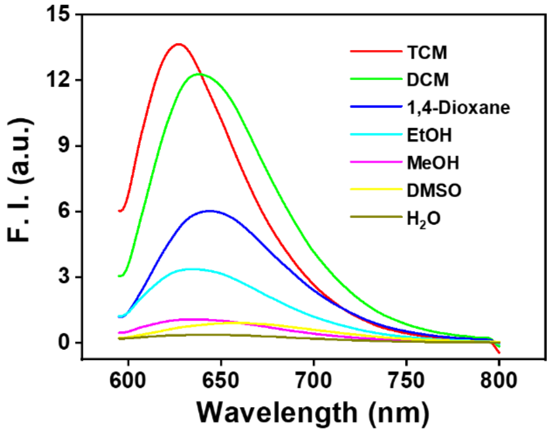

Figure S43. Fluorescence emission spectra of MP-RHO in different solvents. DCM: dichloromethane;

TCM: trichloromethane; MeOH: methanol; EtOH: ethanol; DMSO: dimethylsulfoxide. Concentration = 1.7×10^{-5} M; λ_{ex} = 575 nm.

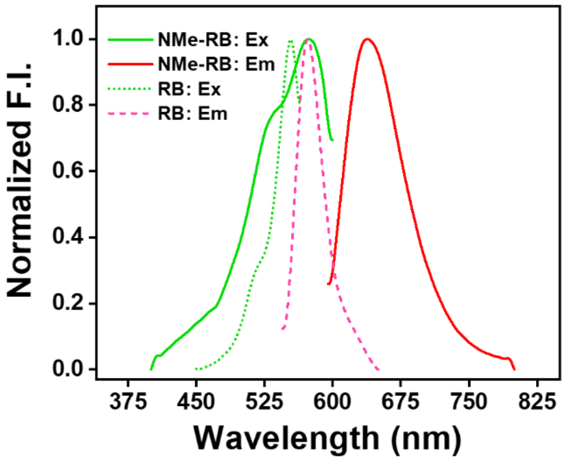

Figure S44. Fluorescence excitation (green) and emission (red) spectra of DEA-RHO (dashed lines) and MP-RHO (solid lines) in dichloromethane. Concentrations of the compounds = 2.0×10^{-5} M. All measurements for MP-RHO were conducted in the presence of 0.1% (*v*/*v*) trifluoroacetic acid. DEA-RHO: λ_{ex} = 550 nm; λ_{em} = 570 nm. MP-RHO: λ_{ex} = 575 nm; λ_{em} = 630 nm.

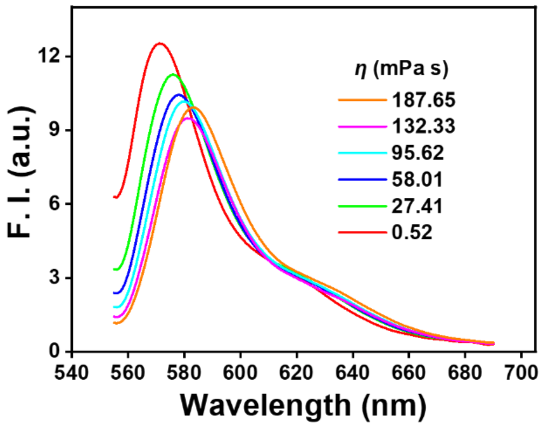

Figure S45. Fluorescence emission spectra of DEA-RHO in the mixtures of methanol and glycerol with different viscosities. Concentration = 2.0×10^{-5} M; λ_{ex} = 550 nm.

7. Photostability of *N*-methylpyrrole substituted fluorophores

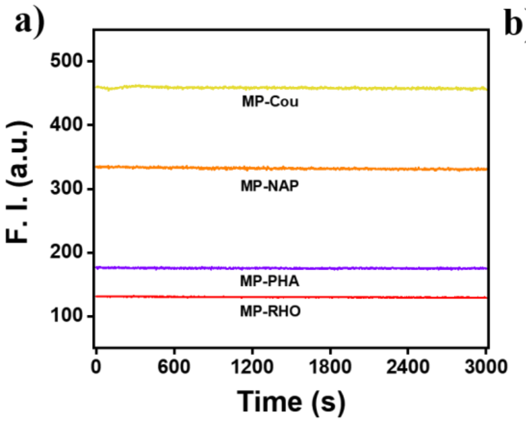

b)

Compound	I_0	I	$(I_0-I)/I_0 \times 100\%$
**MP-COU**	459.93	455.47	0.96
**MP-NAP**	334.07	331.57	0.75
**MP-PHA**	176.74	174.78	1.10
**MP-RHO**	131.21	128.67	1.93

Figure S46. Photostability of the N-methylpyrrole probes. (a) Plots of fluorescence intensity of the fluorophores *vs* continuous irradiation time at the maximum excitation wavelength of each fluorophore in dichloromethane. (b) Calculated photobleaching efficiency for each fluorophore. Irradiation wavelength/monitoring wavelength: MP-NAP (400/530 nm); MP-PHA: (375/525 nm); MP-COU: (400/510 nm); MP-RHO: (575/630 nm). Concentration of the probes = 2.5×10^{-5} M.

8. Albuminuria detection in artificial urine

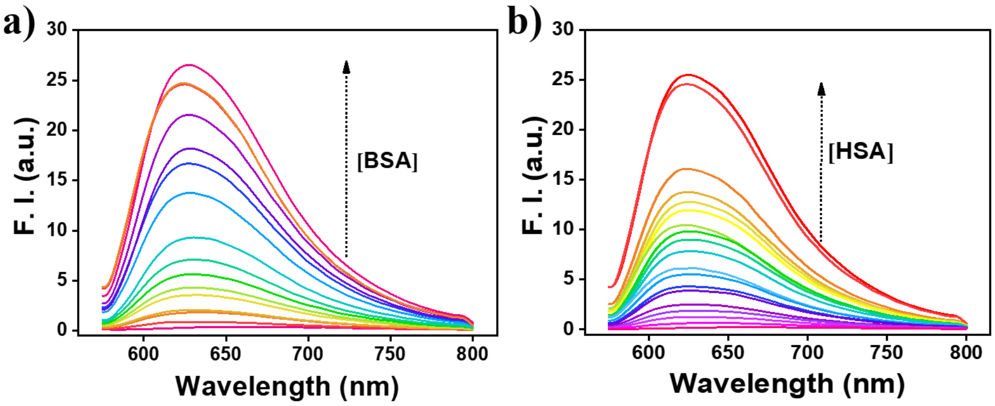

Figure S47. Fluorescence emission spectra of MP-RHO in PBS with different concentrations of BSA (a) and HSA (b). The measurements were conducted in the presence of 0.1% (*v*/*v*) trifluoroacetic acid. Concentration of MP-RHO = 1.8×10^{-5} M; λ_{ex} = 575 nm.

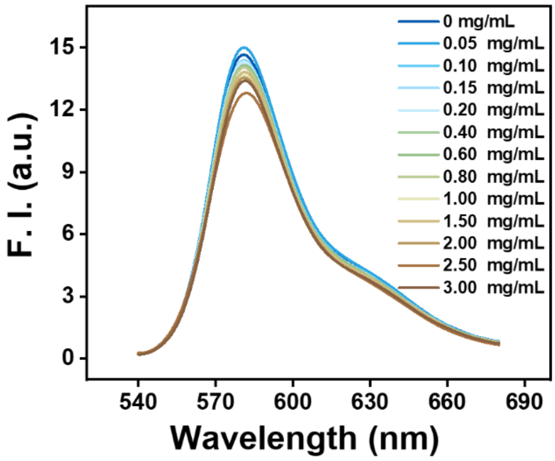

Figure S48. Fluorescence emission spectra of DEA-RHO in urine (pH = 4.7) with different amounts of HSA. Concentration = 1.3×10^{-5} M; λ_{ex} = 550 nm.

9. References

[1] R. Miao, D. Wang, J. Xiao, J. Ma, D. Xue, F. Liu, Y. Fang, *Phys. Chem. Chem. Phys.* **2020**, *22*, 10212-10218.

[2] C. A. Hoelzel, H. Hu, C. H. Wolstenholme, B. A. Karim, K. T. Munson, K. H. Jung, H. Zhang, Y. Liu, H. P. Yennawar, J. B. Asbury, X. Li, X. Zhang, *Angew. Chem. Int. Ed.* **2020**, *59*, 4785-4792.

[3] J. Chen, C. Wang, W. Liu, Q. Qiao, H. Qi, W. Zhou, N. Xu, J. Li, H. Piao, D. Tan, X. Liu, Z. Xu, *Angew. Chem. Int. Ed.* **2021**, DOI: 10.1002/anie.202111052.
